# Pangenomics reveals alternative environmental lifestyles among chlamydiae

**DOI:** 10.1038/s41467-021-24294-3

**Published:** 2021-06-29

**Authors:** Stephan Köstlbacher, Astrid Collingro, Tamara Halter, Frederik Schulz, Sean P. Jungbluth, Matthias Horn

**Affiliations:** 1 Centre for Microbiology and Environmental Systems Science, University of Vienna, Vienna, Austria; 2 DOE Joint Genome Institute, Berkeley, CA USA; 3 Present Address: Laboratory of Microbiology, Wageningen University and Research, Wageningen, The Netherlands

**Keywords:** Microbial ecology, Metagenomics, Symbiosis

## Abstract

Chlamydiae are highly successful strictly intracellular bacteria associated with diverse eukaryotic hosts. Here we analyzed metagenome-assembled genomes of the “Genomes from Earth’s Microbiomes” initiative from diverse environmental samples, which almost double the known phylogenetic diversity of the phylum and facilitate a highly resolved view at the chlamydial pangenome. Chlamydiae are defined by a relatively large core genome indicative of an intracellular lifestyle, and a highly dynamic accessory genome of environmental lineages. We observe chlamydial lineages that encode enzymes of the reductive tricarboxylic acid cycle and for light-driven ATP synthesis. We show a widespread potential for anaerobic energy generation through pyruvate fermentation or the arginine deiminase pathway, and we add lineages capable of molecular hydrogen production. Genome-informed analysis of environmental distribution revealed lineage-specific niches and a high abundance of chlamydiae in some habitats. Together, our data provide an extended perspective of the variability of chlamydial biology and the ecology of this phylum of intracellular microbes.

## Abstract (web summary)

Chlamydiae are strictly intracellular bacteria that exist in a wide variety of environments but the diversity of the phylum is not well described. Here, the authors analyze 82 metagenome-assembled genomes, identify seven new families, and describe genomic signals of metabolic diversity.

## Introduction

Microbes specialized to live inside eukaryotic cells are diverse and have emerged independently among various bacterial and archaeal taxa. This includes pathogens of humans as well as beneficial symbionts of animals, overall with a major impact on the life around us^1^. Intracellular bacteria are generally studied in the context of a particular host, e.g., with respect to a disease or nutritional interactions, and focused on groups of closely related microorganisms. One of the most diverse, successful, and ancient bacterial lineages intimately associated with eukaryotes is the phylum Chlamydiae^2,3^. Studying these microbes has the potential to understand the variability and evolution of the intracellular lifestyle in a much broader context, across an array of different eukaryotic hosts, environments, and over extended evolutionary time scales.

The Chlamydiae are part of the Planctomycetes-Verrucomicrobia-Chlamydiae (PVC) superphylum, a group that, apart from Chlamydiae, predominantly consists of free-living bacteria of environmental and biotechnological importance^4,5^. Chlamydiae were long thought to consist of a single family, the Chlamydiaceae, including several well-known human and animal pathogens^3,6^. Yet, molecular diversity surveys suggest the existence of hundreds of chlamydial families in a great range of different environments^7,8^. Our knowledge about these microbes, commonly referred to as environmental chlamydiae^2^, is sparse, except that many of them are likely associated with protist hosts^7,9^. These unicellular eukaryotes are ubiquitous and make up more than twice the biomass on earth than all animals combined^10^. However, the isolation and cultivation of chlamydiae is challenging and was so far only successful for members of six chlamydial families^7,11^. Confounding factors include their strict dependence on eukaryotic host cells, the fact that the natural host is often unidentified^11^, and unknown growth conditions aggravating the cultivation of protists. Despite the phylum-level diversity of chlamydiae, their intracellular lifestyle appears to be well-conserved as all cultured representatives share a unique developmental cycle consisting of alternation between an infectious extracellular stage, the elementary body (EB), and an intracellular replicative stage, the reticulate body (RB)^6^.

In the face of the experimental challenges associated with the intracellular lifestyle and for a long time the lack of methods to genetically modify chlamydiae^12^, genomics of cultured representatives has been of particular importance to understand chlamydial biology and host interaction^13–16^. Recent advances in metagenomics and single cell genomics enabled the recovery of single cell amplified genomes (SAGs) and metagenome-assembled genomes (MAGs) from yet uncultured chlamydiae despite their generally low abundance in complex microbial communities^16–22^. This revealed a number of surprising findings and provided a first glimpse at the genomic versatility of environmental chlamydiae^18,19^. For instance, marine chlamydial SAGs encoded a complete flagellar apparatus, while all known chlamydiae are non-motile^18^. Furthermore, chlamydial MAGs were strikingly abundant in anoxic marine deep sea sediments^19,23^. This was particularly unexpected as chlamydiae had been considered aerobic or microaerobic microbes. In contrast, the anoxic sediment MAGs showed features indicative for an anaerobic metabolism^19,23^. Previous studies have consistently described hundreds of genes conserved in all or nearly all members of the phylum Chlamydiae^15,16,20^, denoting a large core genome^24^. The accessory genome, i.e., the set of genes encoded only in one or few representatives, indicates potential niche or host-specific adaptations and seems to be expanded in environmental chlamydiae—although comprehensive analyses are missing so far. More generally, the pangenome, i.e., the sum of core and accessory genome, can give insights into habitat specificity and evolutionary forces shaping microbial genomes^24^.

Here we used the Chlamydiae as a model to study the variability of the intracellular lifestyle in the context of an entire bacterial phylum and a global genome sequence dataset. To this end, we capitalized on the Genomes from Earth’s Microbiomes (GEM) initiative, which represents a comprehensive collection of MAGs from diverse environments worldwide^25^ (https://genome.jgi.doe.gov/GEMs). Our analysis of chlamydial MAGs from this resource expands recognized chlamydial taxonomic richness based on genomic data by almost doubling representatives at the species and genus rank. We discovered additional chlamydial families and provide evidence for surprisingly widespread distribution of the potential for anaerobic metabolism as well as a number of other niche-specific adaptations. Genome-informed mining of public 16S ribosomal RNA (16S rRNA) gene data revealed distinct and lineage-specific environmental preferences, with many yet uncultured chlamydiae reaching high abundances and being found in diverse aquatic systems.

## Results and discussion

### A phylogenomic perspective on chlamydial diversity

**Fig. 1:**
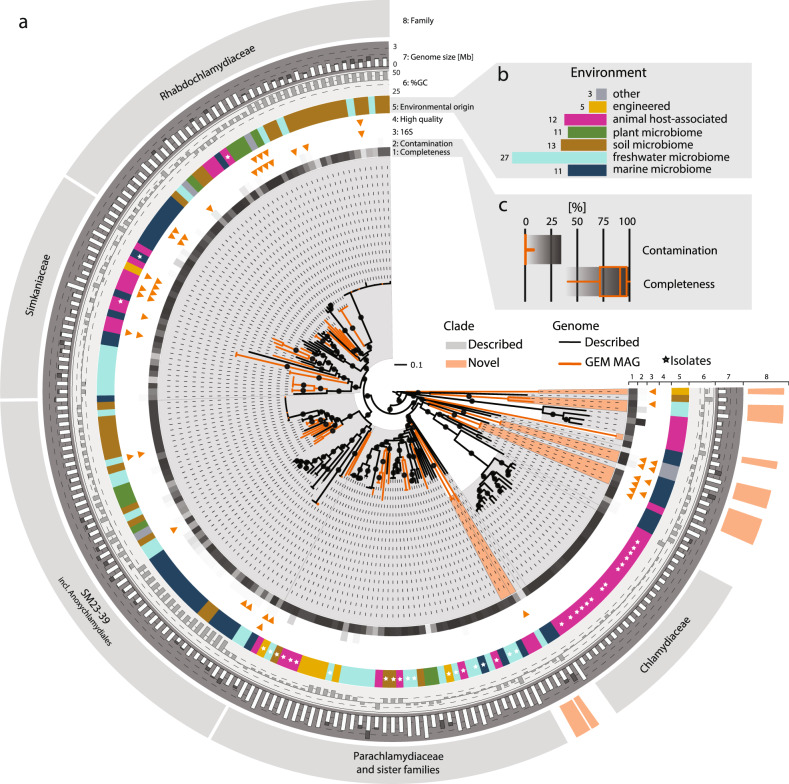
MAGs from diverse environments expand known and add previously undescribed clades in the phylum Chlamydiae. **a** Maximum likelihood phylogenetic tree based on a concatenated set of 43 conserved marker proteins (5704 sites) in which published genomes and 82 MAGs generated in the GEM initiative are shown in black and orange, respectively. Chlamydial monophyly was supported by optimized ultrafast bootstrap and SH likelihood ratio test support with 100% for both. Previously established chlamydial families are shaded in light gray, previously undescribed families are shaded in orange. The tree was inferred under the LG + C60 + G4 + F model with the IQ-TREE software. Nodes with an optimized ultrafast bootstrap support ≥ 95% are labelled with black circles. Tree annotations from inside to outside: (1) completeness, (2) contamination, (3) MAG with 16S rRNA gene, (4) high quality MAGs, (5) environmental origin (white stars indicate genomes from cultured isolates), (6) %GC content, (7) assembly size and estimated genome size (stacked white and gray bars, respectively), and (8) names of chlamydial families represented by more than ten genomes and added metagenomic clades indicated by orange segments. Scale bar indicates 0.1 substitutions per position in the alignment. **b** Number of MAGs retrieved per environmental category. **c** Completeness and contamination estimates of chlamydial MAGs from the GEM dataset. Shaded gradients behind the completeness and contamination boxplots represent the values in the heatmap boxes in the tree.

In total, 82 MAGs from the GEM dataset were classified as members of the phylum Chlamydiae^25^. Phylogenomic analysis using a set of 43 conserved marker proteins confirmed that all MAGs are of chlamydial origin and distributed throughout the chlamydial species tree obtained with a reference dataset including published and few newly determined genome sequences (Fig. 1, Supplementary Data 1 and 2). In line with MIMAG standards (Minimal Information about a Metagenome-Assembled Genome)^26^, 67 MAGs have medium quality corresponding to an estimated genome completeness over 50% and contamination lower than 10%. The remaining 15 MAGs are high quality with an estimated completeness of over 90%, contamination under 5%, a full-length 16S rRNA gene, and more than 18 tRNAs (Fig. 1, Supplementary Data 1).

Consistent with known chlamydial genomes, the 82 MAGs show a reduced (estimated) genome size (0.9–2.6 Mb, average 1.6 Mb) and a moderately low average GC content (42.6%, range 25.9–49.8%; Fig. 1, Supplementary Data 1). In general, chlamydiae associated with multicellular eukaryotes have smaller genomes, while chlamydial symbionts of protists show larger genome sizes^15,19^. The MAGs from this study might thus represent both animal and protist-associated chlamydiae.

Based on our de novo species tree (Fig. 1a), we estimated the level of taxon sampling in the chlamydiae by calculating phylogenetic diversity and phylogenetic gain, representing the sum of branch lengths in the tree and the added branch lengths by a group of taxa, respectively^27^ (Supplementary Data 3). The added MAGs represented 39.5% of the total branch length in the chlamydial species tree, thus almost doubling the known chlamydial phylogenetic diversity.

Next, we inferred the environmental origin of the MAGs using metadata from the Integrated Microbial Genomes and Microbiome database IMG/M^28^ supplemented by additional information from the literature (Fig. 1 and Supplementary Data 4). More than two-thirds of the MAGs are derived from aquatic sources and terrestrial habitats (*n* = 38 marine and freshwater microbiomes; *n* = 24 soil and plant microbiomes), further supporting a ubiquitous occurrence of chlamydiae in the environment^7^. These findings reflect 16S rRNA gene based studies, suggesting marine, freshwater, soil, and plant systems as environmental reservoirs of chlamydiae^8,29^. Most of the additional diversity observed here is due to MAGs from freshwater and marine environments (19.2% and 10.9% phylogenetic gain, respectively; Supplementary Data 5). Soils add only 2.5% phylogenetic gain, indicating that this environment already has been well sampled with respect to chlamydial diversity. Notably, the second highest total phylogenetic gain (18.1%) was obtained from MAGs detected in host-associated animal microbiomes (Supplementary Data 1). There are no known chlamydiae infecting plants^7,30,31^, and consistent with this, MAGs from plant microbiomes were mostly derived from rhizosphere, rhizoplane, and phyllosphere samples, with the exception of three MAGs originating from surface-sterilized *Populus* roots, i.e., the endosphere^32^.

### Metagenomics-driven discovery of taxa

**Fig. 2:**
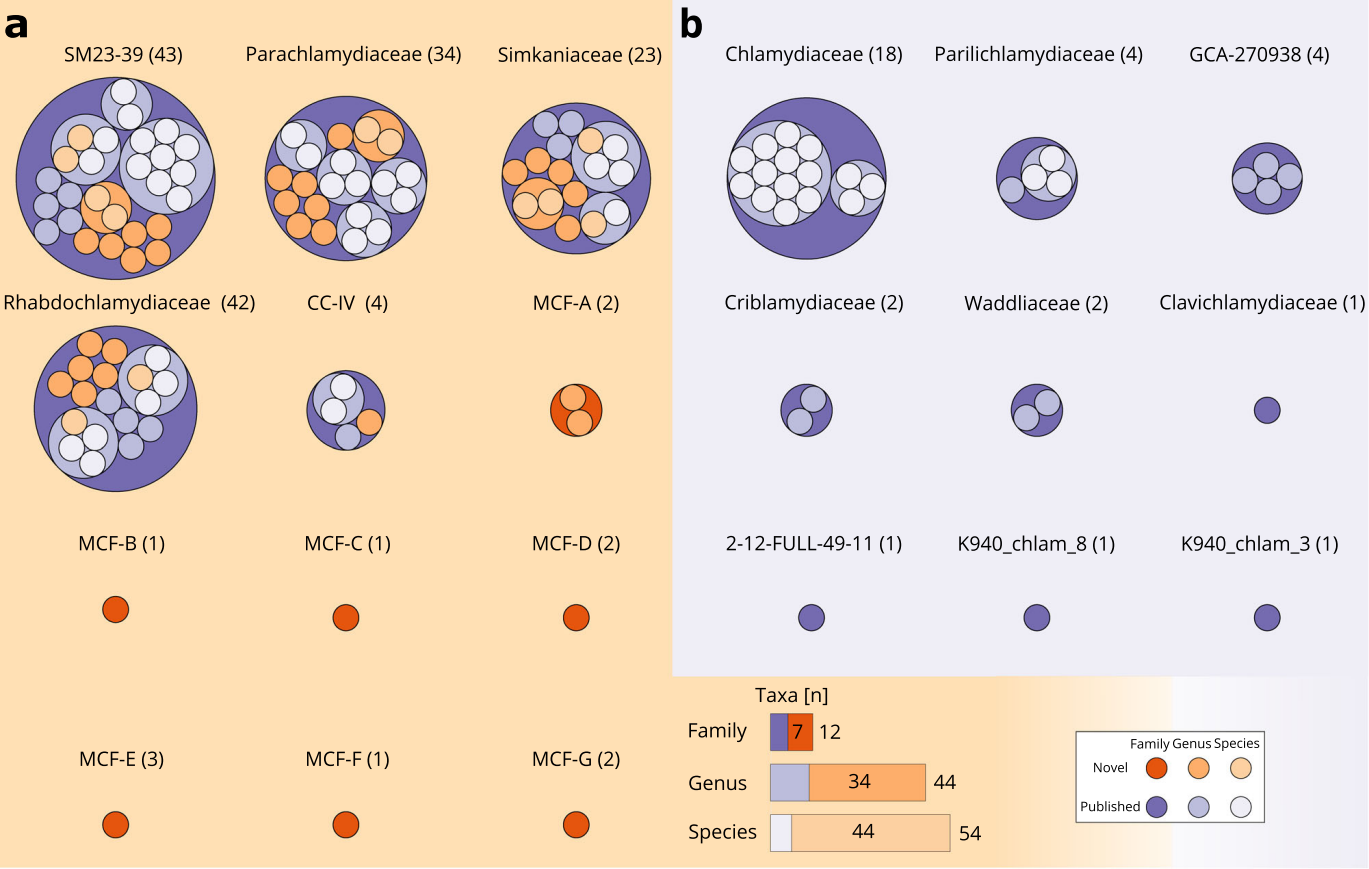
MAGs from the GEM dataset broadly populate the taxonomy of the Chlamydiae at family-, genus-, and species rank. MAGs from the GEM catalog significantly extend known chlamydial taxa, including 7 additional families, 34 genera, and 44 species, highlighting the taxonomic heterogeneity of the phylum. Packed circles represent chlamydiae taxonomic ranks and their higher level taxonomic structure. From the outermost to the innermost circle the family, genus, and species ranks are depicted. Violet indicates lineages with previously known genome representatives (family, genus, species rank in dark, medium, and light violet, respectively), while added lineages are shown in orange (family, genus, species in dark, medium, and light orange, respectively). The number in brackets next to the family names indicates the number of genome sequences available. **a** Known and previously undescribed chlamydial families containing MAGs from the GEM catalog. Bar charts represent the number of families, genera, and species recruited in this study. **b** Families without genome sequences from this study.

To assign chlamydial MAGs to taxonomic units, we used the relative evolutionary distance (RED) approach of the Genome Taxonomy Database GTDB^33^. We classified all MAGs with the GTDB-tk toolkit and used our de novo species tree as additional reference and for refinement, as the GTDB framework only allows classification to known taxa in the database. Sixty-nine MAGs were assigned to five existing chlamydial families. All were confirmed by our species tree except for three MAGs (1039677-28, 1039689-34, and 1039701-25), which represented a highly supported sister clade to the GTDB family GCA-270938. Consistent with this grouping RED values indicated that the three MAGs establish a separate family, for the purpose of this study referred to as Metagenomic Chlamydial Family MCF-E (Supplementary Data 7). In total, 13 MAGs represent seven previously undescribed family-rank clades, derived mostly from aquatic environments and denoted here as MCF-A to MCF-G (Fig. 2; Supplementary Data 1, 6, and 7; families MCF-D and MCF-E represented by high quality MAGs, the other families by medium quality MAGs according to MIMAG standards).

To better understand the taxonomic diversity within chlamydial families, we used a whole genome average nucleotide identity (ANI) and average amino acid identity (AAI) based clustering to resolve the species and genus rank, respectively (Supplementary Figs. 1–3, Supplementary Data 4). We identified 54 species in 44 genera among the 12 chlamydial families that contained the MAGs from the current study. The GEM dataset comprises more previously unknown than described chlamydial taxa on all taxonomic ranks analyzed, including 44 species and 34 genera (Fig. 2). Notably, the highest number of added genera was found in families whose members are traditionally considered environmental representatives of the phylum, often associated with amoeba or arthropods. This includes the Parachlamydiaceae, the Simkaniaceae, and the Rhabdochlamydiaceae (in GTDB v89 named Ga0074140) (Fig. 2, Supplementary Data 7). In addition, the recently described family SM23-39 (also referred to as Limichlamydiaceae or Anoxychlamydiales)^17,19,20^, so far represented by MAGs exclusively, includes seven additional genera.

In total, after the addition of the MAGs from the GEM catalog, 117 chlamydial species, 94 genera, and 21 families are currently represented with genomic data, leading to an increase of 60%, 57%, and 50%, at the respective taxonomic rank (Fig. 2; Supplementary Data 7). Our analysis thus corroborates the large chlamydial diversity estimates inferred from 16S rRNA gene surveys. The additional genome data provides an important step toward understanding chlamydial diversity in the environment.

### A stable lifestyle-reflecting core genome and genomic plasticity in environmental lineages

Genes shared across all genomes of a set of organisms, also referred to as the core genome, provide evidence for conserved biological features^24^. We de-replicated all 192 genomes of our dataset at 99% ANI to reduce redundancy and only included genomes that were the most complete (≥85%) and the least affected by contamination (≤5%). This resulted in a representative dataset for the phylum of 96 genome sequences (Supplementary Data 8). We next inferred non-supervised orthologous groups (NOGs) corresponding to gene families represented in the dataset^34,35^. 375 NOGs were conserved among more than 90% of all genomes, forming the chlamydial core genome (Supplementary Fig. 4). This amounts to a median of 25% of NOGs per genome (interquartile range 22–34%). The core genome size is thus in the range of early estimates including genomes of only ten cultured representatives (*n* = 560, core genome: conserved in all genomes)^15^, and more recent analyses including a few MAGs and SAGs (*n* = 108; no quality filtering; core genome: conserved >90% of genomes)^20^, or genomes of six chlamydial families, respectively (*n* = 342; core genome: conserved in all genomes)^16^.

**Fig. 3:**
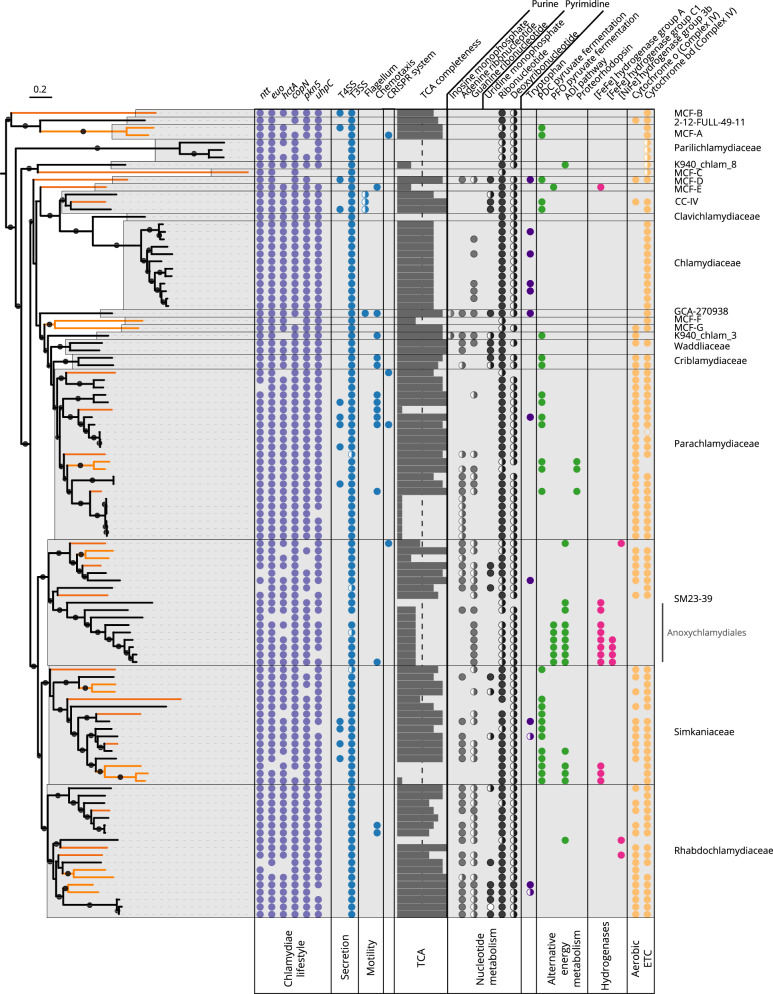
Chlamydiae show conserved features of an intracellular lifestyle but versatility in oxygen adaptation. The presence of selected genes and pathways across the chlamydial core and accessory genome is depicted. The phylogenetic tree includes 96 high quality genomes used for pangenome analysis and additional representatives (*n* = 109 in total). The tree is based on a concatenated set of 43 conserved marker proteins (6268 sites) and was inferred under the LG + C60 + G4 + F derived PMSF approximation by the IQ-TREE software. Branch support values are based on 100 non-parametric bootstraps, support ≥ 70% is indicated as black circles. MAGs from the GEM catalog are indicated by orange branch colors. Colored circles show full or partial presence of selected genes or metabolic pathways. Pyruvate fermentation refers to the presence of the full pathway for pyruvate fermentation to acetate and is differentiated based on the presence of the enzyme for acetyl-CoA generation from acetate, i.e., pyruvate dehydrogenase complex (PDC), or pyruvate ferredoxin oxidoreductase (PFO) together with phosphate acetyltransferase and acetate kinase, or acetate-CoA ligase. The arginine deiminase (ADI) pathway is only indicated if arginine deiminase, ornithine carbamoyltransferase, and carbamate kinase were found. Bar chart shows the completeness of the tricarboxylic acid cycle (TCA). Genes encoding nucleotide transport proteins (*ntt*); early upstream ORF (*euo*), transcriptional regulator of the chlamydial developmental cycle; histone-like developmental protein (*hctA*); serine/threonine protein kinase CopN (*copN*); pseudokinase Pkn5 (*pkn5*); glucose 6-phosphate transporter (*uhpC*); type IV secretion system (T4SS); type III secretion system (T3SS).

The chlamydial core genome determined with our dataset encompasses a number of recognized features facilitating the lifestyle of known chlamydiae and representing hallmarks of intracellular microbes (Fig. 3). This includes the non-flagellar type III secretion system (T3SS), a key virulence mechanism translocating effector proteins into chlamydial host cells^15,19,36,37^. Effector proteins interfere with host cellular pathways as exemplified by the conserved serine/threonine protein kinase CopN (present in 99% of dereplicated genomes)^38^ and the pseudokinase Pkn5 (93%)^39^. All known chlamydiae rely on host-derived metabolites^40^. Our analysis suggested that glucose-6-phosphate can likely be scavenged by all chlamydiae using the glucose 6-phosphate transporter UhpC (97%, only missing in one MAG and two draft genomes)^41^. In addition, the core genome includes a suite of nucleotide transport proteins (98%, Ntp1; 96%, Ntp2) to import ATP and other nucleotides^42–44^. Of note, the master regulator of the unique chlamydial developmental cycle, EUO, is highly conserved (99%)^45^. To a lesser degree, this is also the case for the histone-like protein HctA (83%), which facilitates the conversion of RBs to the EB stage^46^ (Fig. 3). Taken together, the chlamydial core genome includes both hallmarks of a conserved developmental cycle and an host-associated lifestyle.

Zooming in from the phylum level to the family rank, we next set out to investigate the pangenome of selected chlamydial families. Calculating the core genome for families with at least three members (Supplementary Fig. 5a), we retrieve a median size of 599 NOGs per family, which is considerably larger than the phylum core genome. Furthermore, the presence-absence hierarchical clustering of core NOGs reflected the grouping of chlamydial families in our phylogenetic analysis, together indicating selection of family-level traits over extended evolutionary time periods (Fig. 3, Supplementary Fig. 5b). The fish pathogenic Parilichlamydiaceae have the smallest core genome with 415 NOGs. They also show the so far most reduced chlamydial genomes with estimated genome sizes < 1 Mb and a pronounced reduction in metabolic capacity (Fig. 3; Supplementary Fig. 5)^16^. In comparison, the protist-associated Parachlamydiaceae have a core genome of 727 NOGs (Fig. 3; Supplementary Fig. 5a), in line with their larger genomes and generally more complete metabolic capabilities^15,40^. Genes that do occur in only some but not all genomes of a group of organisms are together referred to as accessory genome, often comprising niche or organism-specific features^24^. Prominent examples of the chlamydial accessory genome are the patchy nucleotide and amino acid synthesis pathways, and the variations in the tricarboxylic acid cycle (TCA) observed in our dataset (Fig. 3, Supplementary discussion 1, Supplementary Data 9).

The relative contribution of core and accessory genes to the pangenome can provide insights into genome evolution^24^. Such analysis is, however, inherently prone to differences in sample size, i.e., the number of available genomes per family. We therefore included only families represented by at least ten genomes and chose to analyze the genomic fluidity parameter, which was shown to be robust to small sample sizes^47^ (see Methods). The parameter measures the dissimilarity of genomes at the gene level within a taxonomic rank by averaging the dissimilarity of genomes within this group—denoted as mean *φ*, where 0 means highly similar and 1 indicates dissimilar genomes, respectively^47^. We focused our analysis on the family pangenomes of the human and animal pathogens in the Chlamydiaceae and the protist-associated Parachlamydiaceae. As expected for a highly specialized intracellular pathogen like *Chlamydia trachomatis*, the Chlamydiaceae showed a low genomic fluidity, as their genomes are highly similar at the gene level (mean *φ* = 0.1; 41% core genes). This is consistent with *Chlamydia trachomatis* having a closed species pangenome, indicating generally small population sizes and limited impact of horizontal gene transfer (HGT)^48^. The protist-associated Parachlamydiaceae, on the other hand, showed a significantly more open pangenome compared to the Chlamydiaceae (mean *φ* = 0.5; false discovery rate adjusted *p* value of *t*-test < 0.001; 6% core genes; Supplementary Fig. 6). This suggests that genome evolution of members of these environmental chlamydiae was characterized by larger population sizes and more interactions with other microbes, e.g., through a larger host spectrum and contact to other (facultative) intracellular microbes within their environmental hosts. This might have facilitated adaptive evolution through HGT, which is consistent with the concept of protists as “melting pots” for the evolution of intracellular bacteria^49,50^. In line with the Parachlamydiaceae, all other chlamydial families that could be included in this analysis also showed open pangenomes, suggesting a great genotypic and phenotypic plasticity across several chlamydial clades (Supplementary Fig. 6).

### Clade-specific potential for inorganic carbon fixation and light-driven ATP synthesis

Some environmental chlamydiae encode features that deviate from the generally highly conserved biology of the majority of known chlamydiae. Among these are gene sets for a flagellar apparatus and a chemosensing system^15,18,19,51,52^, a conjugative type IV secretion system^15,53^, and the CRISPR-Cas phage defense system^54,55^. We recovered all of these features in our extended genome dataset and found support for a more widespread occurrence among different chlamydial lineages (Fig. 3; Supplementary discussion 2, Supplementary Fig. 7, Supplementary Data 10 and 11).

The MAGs from the GEM catalog added 45% novel gene families (NOGs) to our dereplicated and quality filtered dataset—gene content that has not been associated with chlamydiae before. Among these, an unexpected finding was the presence of key enzymes of the reductive tricarboxylic acid cycle (rTCA), a pathway for carbon fixation in microoxic and anaerobic microbes. We detected genes encoding ATP-citrate lyase (AclA and AclB) in MAGs of the MCF-D family from antarctic saline lakes (Fig. 3, Supplementary Data 9)^56^. Based on the AclA phylogeny, the chlamydial enzyme is related to ATP-citrate lyases from Epsilonproteobacteria and Aquificae (Supplementary Fig. 8). Host-associated microbial photo- or chemoautotrophic carbon fixation is important in many marine invertebrates^57,58^, yet the chlamydial MAGs lack the full potential for photo- or chemoautotrophy (i.e., the ferredoxin-dependent pyruvate synthase and the 2-oxoglutarate synthase). The partial rTCA in these chlamydiae might instead function in a similar fashion as in the pathogen *Mycobacterium tuberculosis*, which uses the pathway to maintain proton gradient and red-ox balance for short-term survival of hypoxia^59^.

**Fig. 4:**
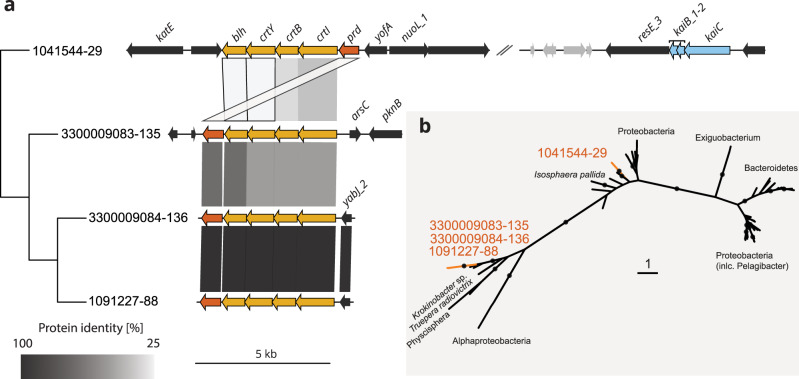
Independent acquisition of light-driven ATP synthesis in two potentially amoeba-associated clades. **a** Gene synteny plot of proteorhodopsin related gene clusters in Parachlamydiaceae MAGs. Comparisons are ordered according to the phylogenomic species tree in Fig. 1. Arrows colored in orange, yellow, and blue represent proteorhodopsin (*prd*), carotene biosynthesis, and circadian clock genes, respectively. Black arrows indicate genes with chlamydial homologs. Bands connect homologs and are colored according to their protein identity. All other proteins of contigs encoding proteorhodopsin gene clusters were blasted against the NCBI non-redundant (nr) database to confirm the chlamydial origin of the contig. **b** Maximum likelihood phylogenetic tree of proteorhodopsin (Prd) (ENOG4105CSB) with chlamydial sequences showing two distinct clades. Maximum likelihood tree was inferred under LG + C30 + G + F model with 1000 improved ultrafast bootstraps and 1000 replicates of the SH-like approximate likelihood ratio test. Filled circles at nodes indicate a bootstrap support ≥ 95%. Scale bar indicates the number of substitutions per site.

Previously unknown Parachlamydiaceae genomes revealed evidence for light-driven ATP synthesis in chlamydiae. A member of the genus *Neochlamydia* from a wastewater bioreactor and three novel MAGs of a genus from microbial mats from antarctic freshwater lakes^60,61^ encoded a complete proteorhodopsin gene cluster including enzymes for synthesis of the light-harvesting co-factor retinal (Figs. 3 and 4a). Phylogenetic analysis suggests the independent acquisition of the gene set in two chlamydial lineages, indicating lineage-specific adaptations (Fig. 4b), which is consistent with this trait known to being frequently subject to HGT. Proteorhodopsins are commonly found in marine microbes in the sunlit (euphotic) zone and represent a major mechanism for light-driven ATP synthesis in these systems^62,63^. A marine *Vibrio* strain that gained proteorhodopsin through HGT showed increased long-term survival under resource-limited conditions^64^. It is therefore tempting to speculate that proteorhodopsin in chlamydiae may function as a maintenance mechanism for EBs, prolonging extracellular survival and increasing the chance to encounter new protist hosts. Alternatively, proteorhodopsin-driven energy generation might alleviate the host cell burden during intracellular replication. Taken together, these findings demonstrate that our understanding of chlamydial biology is far from complete, not only with respect to only recently recognized lineages but even for those environmental chlamydiae with cultured representatives.

### Widespread anaerobic and molecular hydrogen metabolism among chlamydiae

**Fig. 5:**
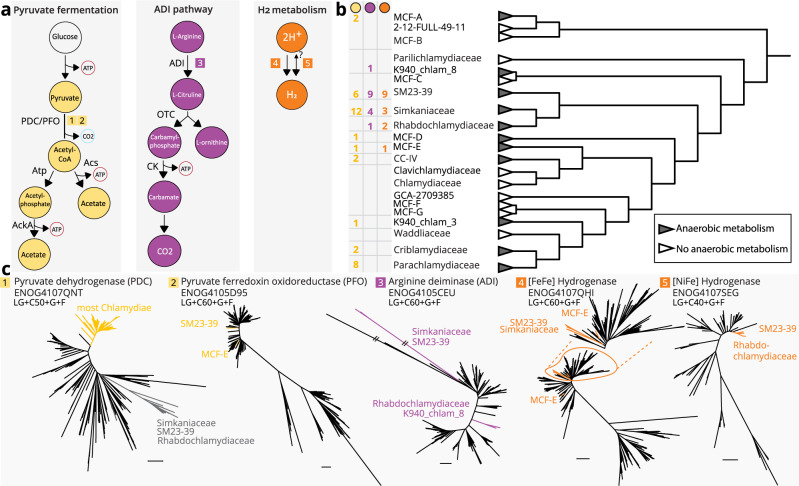
Widespread fermentation pathways and molecular hydrogen production in chlamydiae. **a** Representation of putative anaerobic pathways for ATP generation and molecular hydrogen metabolism in chlamydiae. Labels next to enzymatic reactions indicate the associated enzymes. Numbers in squares correspond to phylogenetic trees in (**c**). Colors indicate affiliation with different pathways—pyruvate fermentation (yellow), ADI pathway (violet), hydrogen metabolism (orange). **b** Species tree of chlamydial representative genomes as in Fig. 3 collapsed at the family rank. Branch support values are based on 100 non-parametric bootstraps, support ≥ 70% is indicated as black circles. Box next to family names indicates the number of non-redundant genomes in a family with the respective color coded metabolic pathway. Pyruvate fermentation to acetate was only counted if genes encoding the complete pathway were present, i.e., pyruvate dehydrogenase complex (PDC), or pyruvate ferredoxin oxidoreductase (PFO) together with phosphate acetyltransferase (Atp) and acetate kinase (AckA), or acetate-CoA ligase (Acs). Likewise, arginine deaminase (ADI) pathway was only counted if genes encoding arginine deaminase (ADI), ornithine carbamoyltransferase (OTC), and carbamate kinase (CK) were present in a genome. **c** Unrooted maximum likelihood phylogenetic trees with best fit models numbered and colored according to (**a**) with 1000 optimized ultrafast bootstrap and 1000 SH-like approximate likelihood ratio test support. Best fit models per gene are indicated under the gene name and clades are named by family.

Chlamydial metabolism has long been understood as aerobic or microaerobic using substrate-level phosphorylation in combination with oxidative phosphorylation^15,40,65^. Yet, recent metagenomic findings in marine deep sea sediments have uncovered a clade of chlamydiae with a specialized anaerobic lifestyle^19,23^. The Anoxychlamydiales (family SM23-39) have the genetic potential to carry out acetogenic fermentation and use the arginine deiminase (ADI) pathway to produce ATP^23^. Like other anaerobic microorganisms, these chlamydiae show an incomplete respiratory chain and a truncated TCA cycle^23^ (Fig. 3). In order to investigate the prevalence of the potential for anaerobic substrate-level phosphorylation, we screened all chlamydial genomes for the presence of known anaerobic pathways and classified them using MetaCyc^66^. Of note, we discovered complete pyruvate fermentation to acetate in 43% of all chlamydial families investigated (9 out of 21; Figs. 3 and 5).

Like the Anoxychlamydiales, members of the family MCF-E have the genetic potential to convert pyruvate to acetyl-CoA using pyruvate ferredoxin oxidoreductase (PFO; Fig. 3, Supplementary Data 9). Phylogenetic analysis suggests that the PFO of these chlamydiae has been independently acquired through HGT. This is consistent with MCF-E members using acetate-CoA ligase^67^ for ATP generation from acetyl-CoA as an alternative to phosphate acetyltransferase (Atp) and acetate kinase (AckA) employed in the Anoxychlamydiales.

The most prevalent pathway of acetogenic fermentation among chlamydiae, however, is acetyl-CoA generation from pyruvate via the pyruvate dehydrogenase complex (PDC), followed by ATP generation and acetate production through the concerted action of Atp and AckA. Genes for these key enzymes are found in representatives of seven families, including cultured members of the Criblamydiaceae, Simkaniaceae, and Parachlamydiaceae, as well as in a number of MAGs from the families CC-IV, MCF-A, MCF-D, and K940_chlam_3 (Figs. 3 and 5). Unlike the Anoxychlamydiales and MCF-E, pyruvate-fermenting chlamydiae with PDC (Fig. 5c) also encode respiratory chain complex IV (cytochrome o and/or cytochrome bd; Fig. 3). This complex is typically associated with aerobic metabolism, but the additional presence of fermentation-related enzymes indicates a facultative anaerobic lifestyle^40^. Well-known protist hosts of environmental chlamydiae, such as *Acanthamoeba*, show a preference for low oxygen conditions and have specialized mitochondria for anaerobic ATP generation^68,69^. Chlamydial lineages infecting these protists may encounter anaerobic conditions, in which the ability to ferment could represent a selective advantage.

An alternative means for anaerobic ATP formation is the ADI pathway, in which arginine is converted to ornithine, ammonia, and carbon dioxide, generating ATP. We find the complete ADI pathway—indicated by the presence of ADI, ornithine carbamoyltransferase, and carbamate kinase—in four chlamydial families, including the Anoxychlamydiales (Figs. 3 and 5)^23^. Our phylogenetic analysis of the key enzyme arginine deaminase retrieved two distinct chlamydial clades (Fig. 5c). This suggests a polyphyletic origin of this gene among chlamydiae, which would be consistent with the ADI pathway being subject to frequent HGT^70^.

In the Anoxychlamydiales fermentation is thought to be coupled to hydrogen production, a strategy to dump electrons in the absence of oxygen or alternative electron acceptors also used by other microbes^23,71^. To investigate the potential for hydrogen metabolism among all chlamydiae, we identified putative hydrogenases based on conserved protein domains and classified them with HydDB^72^. We found in total 40 hydrogenases in 34 genomes, classified as [FeFe] hydrogenases or [NiFe] hydrogenases, respectively (Supplementary Data 12).

[FeFe] hydrogenases previously described in Anoxychlamydiales MAGs are also present in the putative anoxic family MCF-E and a lineage of three Simkaniaceae MAGs associated with gutless marine oligochaetes of geographically distant origin (Figs. 3 and 5; Supplementary Data 12)^73^. All three chlamydial groups encode oxygen-sensitive trimeric [FeFe] hydrogenases to synergistically oxidize NADH and ferredoxin to produce molecular hydrogen^74^ (Supplementary Fig. 9). While these hydrogenases are functionally similar, phylogenetic analysis recovers two separate monophyletic clades, suggesting they have been acquired independently (Fig. 5c). One additional [FeFe] hydrogenase is only present in one MCF-E member and is only distantly related to the two other clades (Fig. 5c, Supplementary Fig. 9).

Of note, we also identified oxygen-tolerant hydrogenases in chlamydial genomes. Type 3b [NiFe] hydrogenases are present in two members of the Rhabdochlamydiaceae, and one member of SM23-39. These cytosolic hydrogenases directly couple oxidation of NADPH to hydrogen evolution but might also catalyze the reverse reaction^75^ (Fig. 5a, reverse reaction annotated with a question mark). All chlamydial homologs are monophyletic and group with the methanotroph *Methylacidiphilum infernorum*, a member of the Verrucomicrobia^76^ (Fig. 5c). Some obligate aerobic mycobacteria use these types of hydrogenases under low oxygen conditions when there is a lack of other terminal electron acceptors^71,77^, suggesting a similar function in chlamydiae.

Molecular hydrogen metabolism is a widespread yet often poorly studied feature in pathogenic bacteria and protists, which is often critical for growth and virulence^71,77^, not only for strict anaerobes such as *Clostridium perfringens*^78^ or the parasite *Trichomonas vaginalis*^79^, but also for the microaerophilic *Helicobacter pylori* and the facultative anaerobe *Campylobacter jejuni*^71,77^.

In summary, our findings reveal surprisingly widespread traits of an anaerobic lifestyle among chlamydiae. This includes apparently strictly anaerobic lineages such as the Anoxychlamydiales and MCF-E, as well as putative facultative anaerobes in the Simkaniaceae, Rhabdochlamydiaceae, Criblamydiaceae, Parachlamydiaceae, and other families (Figs. 3 and 5b). The patchy distribution of fermentation pathways and hydrogenases indicates a complex scenario for the evolutionary relationship of chlamydiae with oxygen.

### Family-specific habitat preferences

**Fig. 6:**
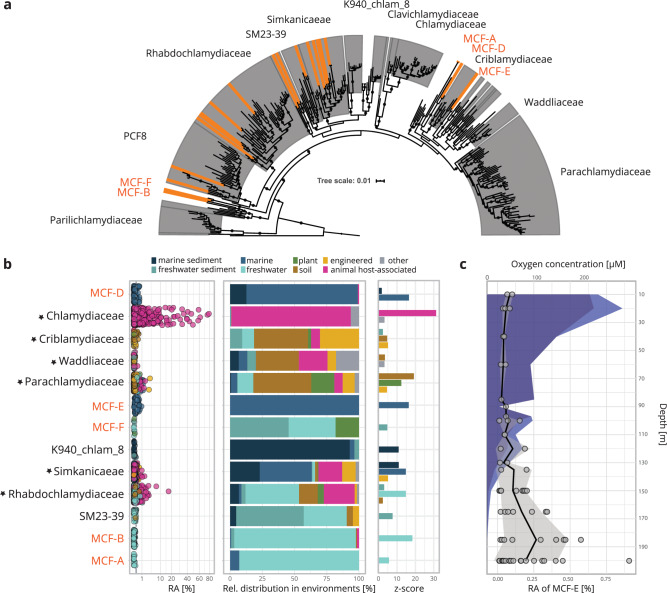
Members of novel chlamydial families predominantly occur in freshwater and marine environments. **a** 16S rRNA gene maximum likelihood phylogenetic tree using near full-length sequences de-replicated at 99% under the SYM + R10 model inferred with IQTREE. Support was inferred from transfer bootstrap expectation (TBE) based on 100 non-parametric bootstraps. Circles at nodes indicate TBE support ≥70. The tree is pruned and does not include the outgroup. Chlamydial families are highlighted by gray background, 16S rRNA genes from novel MAGs in this study are indicated by orange shading. **b** All chlamydial full-length 16S rRNA genes in chlamydial genomes were used as a query against IMNGS with an identity cutoff of 1% to ensure species-specificity and summarized at the family level. Stars next to names indicate families with cultured representatives. Environmental categories “marine” and “freshwater” represent samples originating from the water column. The scatter plot on the left shows the relative abundance of chlamydial 16S rRNA gene amplicons. The bar plot in the middle shows the relative distribution of family members across diverse environments. The bar plot on the right indicates significant enrichment (adjusted *p* value ≤ 0.05) in environments based on one-sided Fisher’s exact test with false discovery rate adjusted *p* values expressed as *z*-scores. **c** Relationship of relative abundance of the anaerobic family MCF-E with oxygen concentration and depth in samples from Saanich Inlet. *Y*-axis depicts depth in meters below surface, top *x*-axis indicates molarity of oxygen and bottom *x*-axis indicates relative abundance in percent of total 16S rRNA amplicons. Dark blue and light blue areas depict mean oxygen concentration and standard deviation, respectively. Gray filled points, black line, and gray area represent relative abundance in a sample, mean relative abundance, and standard deviation, respectively.

We next used our genome sequence dataset to investigate the abundance and distribution of chlamydiae in the environment. Of the chlamydial genomes in this study, 84 of 192 (32/82 MAGs of the GEM catalog) encode near full-length 16S rRNA genes ≥ 1300 nt, covering 15 of 21 chlamydial families with genome representatives. We used these sequences together with all publicly available near full-length 16S rRNA sequences and dereplicated the dataset at 99% sequence identity^80^, yielding 310 chlamydial species representatives. Phylogenetic analysis confirmed the monophyly of all chlamydiae with high support (Fig. 6a), and the 16S rRNA gene tree corroborated the genome-based classification of chlamydial families (Fig. 1). While most sequences from the GEM catalog are part of chlamydial families identified earlier, sequences of the putative families MCF-A, MCF-B, MCF-D, MCF-E, and MCF-F represent yet unrecognized lineages in the 16S rRNA-based tree (Figs. 2 and 6a).

We queried all chlamydial 16S rRNA sequences for which a genome sequence is available against the integrated microbial next-generation sequencing (IMNGS) database^81^ with 99% identity to estimate environmental species-level distribution and abundance. We obtained chlamydial amplicons matching the 16S rRNA gene of genomic representatives from 3,261 samples. Consistent with previous 16S rRNA meta-analysis, chlamydiae can be found in all major environments, as well as in a multitude of eukaryotic microbiomes^7,8^. Presence and relative abundance (RA) information was summarized at the family rank in order to investigate habitat preferences of the major lineages in our genome dataset. We obtained amplicon hits for 13 of the 15 families, only missing the fish pathogens Clavichlamydiaceae and Parilichlamydiaceae. Indeed, members of these families have only been found in fish gills so far^82,83^, for which no public microbiome studies are available (https://www.imngs.org/; April 2020). This suggests that the Clavichlamydiaceae and Parilichlamydiaceae might be limited to these vertebrate hosts and not associated with microbial eukaryotes.

Unsurprisingly, Chlamydiaceae show a highly significant association with animal host-associated microbiomes (Fig. 6b). If present, members of the Chlamydiaceae reach RA values in the bacterial community of up to 79% in a variety of human and animal microbiomes^84–88^ (Supplementary Fig. 10, Supplementary Data 13). For chlamydial families with cultured representatives in protists, we observe significant enrichment in soil (Parachlamydiaceae, Criblamydiaceae, Waddliaceae) and engineered environments (Parachlamydiaceae, Criblamydiaceae, Simkaniaceae), respectively (Fig. 6b), which is coherent with the origin of the majority of isolates from these families.

Families without cultured representatives on the other hand show significant enrichment in marine environments, including MCF-D (water column and sediment), MCF-E (water column), and K940_chlam_8 (sediment). This illustrates that these environments are still undersampled with respect to chlamydiae (Fig. 6b). So far, the Simkaniaceae members *Neptunochlamydia vexilliferae* and *Syngnamydia salmonis* are the only marine isolates available^89,90^. Accordingly, the Simkaniaceae are significantly enriched in marine environments. Even though some evidence for the clinical relevance of the third cultivated representative of this family, *Simkania negevensis*^91^, has been reported^92^, it is found with up to 1.7% RA in coral microbiomes^93^ and at 0.5% RA in anaerobic digesters (Supplementary Fig. 10, Supplementary Data 13). This supports the existence of an environmental niche for *S. negevensis* and corroborates our finding of anaerobic metabolic potential for this and other members of the Simkaniaceae (Figs. 3 and 5).

For family SM23-39, which contains the anaerobic Anoxychlamydiales^17,19,23,94^, the IMNGS query only yielded hits for those members that lack anaerobic pathways or appear to be facultative anaerobes (Fig. 3), and these are enriched in freshwater sediment environments (Fig. 6b, Supplementary Fig. 10, Supplementary Data 13). Owing to the lack of the 16S rRNA gene in all but one Anoxychlamydiales MAG, we could not further assess the environmental distribution of this group. However, the second anaerobic lineage, family MCF-E, is found in marine water column habitats and can reach up to 1% RA (Fig. 6b and 6c, Supplementary Fig. 10, Supplementary Data 13). All 16S rRNA gene sequences from this family originate from samples from Saanich Inlet, a seasonally anoxic fjord at the coast of Vancouver Island, British Columbia, Canada^95,96^. We related RA in samples containing MCF-E amplicons to oxygen concentration and sampling depth and observed the highest abundance of chlamydiae below the oxycline, i.e., in the deeper, anoxic layers of the water column (Fig. 6c). Potential hosts of these chlamydiae are microaerophilic or anaerobic protists, which are known to occur in the anaerobic water column and may, together with methanogenic endosymbiotic bacteria, be important for the biochemical cycling of methane^97^.

In summary, the comprehensive analysis of chlamydial MAGs in this study provides novel insights into the genomic diversity of a bacterial phylum of strictly intracellular microbes, revealing a surprising variation with respect to their biology. Our analysis expands the known phylogenetic diversity of chlamydiae by 40%. We show that the chlamydial core genome comprises the toolbox for an host-associated intracellular lifestyle, while the accessory genome varies strongly in size between different families, reflecting adaptation to various environments and diverse hosts. We found evidence for light-driven ATP synthesis and key genes for the rTCA cycle in chlamydial organisms, and we show that members of several lineages have the genetic potential for anaerobic and hydrogen metabolism. Our genome-informed diversity survey revealed the presence of these chlamydiae in various anaerobic environments and provided further evidence for a ubiquitous occurrence of chlamydiae, sometimes at surprisingly high abundance. Targeted metagenomics and isolation approaches using diverse protist hosts will be important to further investigate those chlamydial groups that are only poorly represented in our datasets so far. Ultimately, this will contribute to a better understanding of how an entire bacterial phylum that engaged in an intracellular lifestyle early on during evolution has emerged, and how niche specialization and adaptation to novel hosts have taken place.

## Methods

### Genome sequencing

The genome sequences of four Parachlamydiaceae symbionts of *Acanthamoeba* spp. isolated from fish gills in Thailand in 2014 have been determined in the context of this study. *Acanthamoeba* isolation and cultivation were carried out as described in Köstlbacher et al.^98^. Briefly, for isolation of genomic DNA amoeba cells were lysed, and host DNA was digested using 10 units DNase I (Thermo Fisher Scientific) at 37 °C for 30 min. DNase digestion was inactivated as recommended by the manufacturer. Bacterial DNA was purified using the DNeasy blood and tissue kit (Qiagen) as recommended by the manufacturer. Sequencing libraries were prepared using the Nextera XT kit (Illumina) and sequenced on an Illumina HiSeq 2000 platform. Trimming and quality control of reads were conducted with BBMap v35.43 (https://sourceforge.net/projects/bbmap/) (bbduk minlen = 50, qtrim = rl, trimq = 25, ktrim = r, k = 25, mink = 11, hdist = 1) and FastQC v0.11.4 (https://www.bioinformatics.babraham.ac.uk/projects/fastqc/). Assemblies were performed with SPAdes v3.5.0^99^, screened for contamination with CheckM^100^, and annotated with prokka v1.14^101^.

### Dataset compilation and quality control

We used 38 MAGs from the published GEM catalog and added 44 MAGs from the GEM project that affiliated with the phylum chlamydiae^25^. Basic MAG features (size, GC content, N50 value, etc.) were calculated with QUAST v5.0.2^102^. Initial gene calling and annotation was performed with prokka v1.14^101^ with the flags“—mincontiglen 200” to call genes only on contigs larger than 200 nt and “—gram neg” for usage of the gram negative database.

In addition to the 82 MAGs of the GEM catalog^25^, we collected all publicly available chlamydial genomes (*n* = 80) on June 25, 2019 from NCBI Genbank and RefSeq, and we added the four Parachlamydiaceae draft genomes, one Rhabdochlamydiaceae MAG from a spider microbiome^103^, one MAG from a metagenome of a marine worm from the genus *Xenoturbella*^104^, and 24 MAGs from deep sea sediment samples^19^ (Supplementary Data 2). As an outgroup, we added 15 non-chlamydial genome sequences of members of the PVC superphylum (Supplementary Data 2). We estimated completeness and contamination of all genomes with CheckM v1.1.2^100^ using general bacterial marker genes with ‘taxonomy_wf domain Bacteria’. We assigned the environmental origin of the genomes based on publicly available metadata. Organisms with known protist hosts were associated with the host environment.

### MAG phylogeny and species tree reconstruction

For comprehensive phylogenomic analysis including all chlamydial MAGs, the protein sequences of 43 conserved marker proteins were extracted and aligned in CheckM v1.1.2 with the ‘tree’ workflow^100^. Model testing and maximum likelihood phylogenies were performed with IQ-TREE 1.6.2^105^ under the empirical LG model^106^. The optimal model was determined with the “-m TESTNEW” procedure^107^. We added the empirical mixture models C10–C60 with the “-madd” option (Best model: C60 + LG + G + F)^108^. Support values were inferred from 1000 ultrafast bootstrap replicates^109^ with the “-bnni” option for bootstrap tree optimization and from 1000 replicates of the SH-like approximate likelihood ratio test^110^. Trees were visualized and edited using the Interactive Tree Of Life v4^111^. We calculated phylogenetic diversity and phylogenetic gain for GEM chlamydiae MAGs in the context of the chlamydial species tree with the GenomeTreeTk v0.1.6 (https://github.com/dparks1134/GenomeTreeTk). We used the MAGs from the GEM catalog as the ingroup and all other chlamydiae were used as outgroup.

To establish a robust species tree, we removed redundancy and low quality MAGs by de-replicating all genomes at 99% ANI with dRep v1.4.3 using default parameters except for “—contamination 10” to remove highly contaminated MAGs. We retained 109 chlamydial and 15 outgroup genomes of the PVC superphylum for downstream analysis^112^. As described above, we calculated a maximum likelihood species tree with IQ-TREE 1.6.2 using the de-replicated dataset (Best model: C60 + LG + G + F). We used this tree as a guide tree for the posterior mean site frequency (PMSF) model^113^ for improved site heterogeneity modeling under the C60 + LG + G + F model and inferred 100 non-parametric bootstraps “-b 100”.

### Taxonomy assignment

Taxonomy was assigned to all genomes with GTDB-Tk v0.3.3^33^ using the ‘classify_wf’ option based on database release version 89 (https://data.ace.uq.edu.au/public/gtdb/data/releases/release89/). Taxonomic novelty for genus and species was inferred based on GTDB. The GTDB-Tk infers RED values for nodes by phylogenetically placing marker protein sequences into the reference species tree (Fig. 1). However, the accuracy of phylogenetic placement decreases with increasing phylogenetic distance^114^. To account for this, we enforced the additional rule that genomes had to be monophyletic (UF-bootstrap ≥ 95%) with the reference genomes at the family rank in the species tree (Fig. 1) in addition to the GTDB assignment. Due to paraphyly with the family rank representative GCA-270938, we therefore changed the taxonomic assignment of the monophyletic group of MAGs 1039677-28, 1039689-34, and 1039701-25 with RED values of 0.70–0.71 (GTDB family novelty below 0.77) to the family MCF-E.

To delineate genus rank clades, we calculated reciprocal best blast hit average amino acid identities (AAI) of chlamydial proteomes as described by Hausmann et al.^115^. We clustered genomes according to Konstantinidis et al.^116^ into genus rank groups at the cutoff of 65% AAI (alignment fraction ≥ 35%). We visualized the density distribution of within family AAI between genomes with the “geom_density” function in the ggplot2 package^117^ (Supplementary Fig. 2). Genus level clusters (AAI ≥ 95% and alignment fraction ≥ 35%) were illustrated with Cytoscape v3.7.0^118^ (Supplementary Fig. 3). Accordingly, we separated species rank clades by calculating the whole genome ANI with FastANI v1.3^119^ and clustering at the 95% ANI cutoff (alignment fraction ≥ 65%). We visualized species-level clusters (ANI ≥ 95% and alignment fraction ≥ 65%) with Cytoscape v3.7.0^118^ (Supplementary Fig. 1). We named previously undetected families, genera, and species according to the MAG with the highest genome quality score (completeness - 5 × contamination) in the respective group. Taxonomic organization of chlamydiae on the family rank and above was performed in R with the ggraph package (https://cloud.r-project.org/package=ggraph).

### Pangenome reconstruction

For pangenome reconstruction, we only considered the 96 genomes of the de-replicated dataset with an estimated completeness > 85% and contamination < 5%. To retrieve orthologous clusters we mapped all protein sequences against eggNOG v4.5.1^34^ with emapper v1.0.1^120^ against the bacterial database “-d bact” and proceeded using these NOGs. We performed an all-against-all blastp search of 45,717 (29.5% of all) unmapped proteins and clustered proteins based on hits with an *E* value < 0.001 with SiLiX^35^ yielding 31,007 de novo NOGs (25,886 singletons). Combining eggNOG and de novo NOGs, the chlamydiae pangenome totaled at 37,380 NOGs (Supplementary Fig. 4). We calculated the chlamydial pangenome subcomponents with the following definitions: core—present in more than 90% of genomes; cloud—present in <15%; and shell—present in 15–90% of genomes^121^. The accessory genome is composed of the cloud and shell genome. We applied the same definitions for family-specific pangenome calculations (Supplementary Fig. 5). The exact numbers of gene families in the accessory genome are dependent on the clustering method and parameters used. However, the general trend of a pronounced difference between Chlamydiaceae and environmental representatives should be largely independent of the thresholds used.

We further analyzed pangenome features for chlamydial families with at least ten genome sequences (Supplementary Fig. 6) to ensure sufficient data points for resampling. We used the micropan^122^ package in R version 3.5.1^123^ genomic fluidity with the “fluidity” function using 100 simulations. We then tested whether the genomic fluidity of Chlamydiaceae is different from other environmental families in this analysis using two-sample *t*-test and corrected for false discoveries using the “p.adjust” function with the “BH”^124^ method in R version 3.5.1^123^.

### Reconstruction of metabolic pathways, and identification of hydrogenases, defense, and secretion systems

We mapped all proteins to Kyoto Encyclopedia of Genes and Genomes (KEGG) orthologs (KOs) using GhostKOALA v2.2^125^. KO associated Enzyme Commission numbers (EC numbers) were used to reconstruct pathways of interest with MetaCyc^66^ or KEGG (Supplementary Data 7). We identified conserved protein domains in all proteins and associated them to metabolic pathways and gene ontology terms using InterProScan v5.35-74.0^126^ with the parameters “-dp—pathways—goterms” using hidden markov models from Pfam^127^, TIGRFAM^128^, and TMHMM^129^ databases. Putative hydrogenases were identified based on conserved TIGRFAM (TIGR02512; [FeFe] hydrogenase, group A) or Pfam domains (PF00374; Nickel-dependent hydrogenase) and verified and classified using the web tool hydDB^72^. Gene synteny plots representing proteorhodopsin or [FeFe] hydrogenase gene clusters in chlamydiae genomes were visualized with genoplotR v0.8.9^130^ (Supplementary Fig. 9). In addition, genomes were screened for the presence of secretion systems and CRISPR cas systems using MacSyFinder v1.0.5^131^ with the “TXSScan” models^132^ with “—db_type ordered_replicon all” and CRISPRCasFinder v2.0.2^133^ with the parameters “-ccc 20000 -ccvRep -html -rcfowce -def S”, respectively. We blasted all identified CRISPR spacers against the viral RefSeq database (https://blast.ncbi.nlm.nih.gov/Blast.cgi) on July 27, 2020.

### Phylogenetic analysis of metabolic genes

For phylogenetic analysis of metabolic genes (including Supplementary Figs. 7 and 8) we downloaded the corresponding NOG protein sequences from eggNOG v4.5.1^34^ and aligned them de novo using mafft v .427 “—maxiterate 1000—localpair”. We trimmed the alignments using BMGE v1.12^134^ using a gap rate of 0.2 “-g 0.2“ and an entropy of 0.6 “-h 0.6”. We calculated maximum likelihood phylogenetic trees with IQTREE v1.6.2^135^ under the empirical LG model^106^ using model testing “-m TESTNEW” including the empirical mixture models C10–C60^108^ and “-seed 12345”. Support values were obtained from 1000 ultrafast bootstraps with bootstrap tree optimization using “-bb 1000 -bnni”^105^ and 1000 replicates of the SH-like approximate likelihood ratio test using “-alrt 1000”^110^. Trees were visualized and edited using the Interactive Tree Of Life v4^111^.

### 16S rRNA gene phylogeny

All available unique near-full length 16S rRNA gene sequences of chlamydiae (*n* = 233) and other PVC members (*n* = 205) were downloaded from SILVA v138 SSU Ref NR 99^136^. An additional 79 near full-length chlamydial 16S rRNA gene sequences (97% identity OTU representatives) from Schulz et al.^29^ were added to the dataset, in addition to 103 sequences from our reference genome dataset totaling 620 near full-length 16s rRNA sequences. Sequences were clustered at 99% sequence identity to reduce redundancy using USEARCH v11.0.667^137^ with “-cluster_smallmem” resulting in 310 Chlamydiae and 198 non-chlamydial PVC members. We aligned the clustered sequences with SINA^138^ and trimmed the alignment with trimAl “-gappyout”^139^. Model testing was performed with IQ-TREE 1.6.2^105^ “-m TESTNEW” (Best model: SYM + R10), and initial support values were inferred from 100 non-parametric bootstraps using “-b 100”. As Felsenstein’s bootstrapping methods tend to yield very low support for large sequence datasets we additionally inferred transfer bootstrap expectation values based on the non-parametric bootstrap trees with booster (https://booster.pasteur.fr/; accessed in April 2020)^140^.

### Environmental distribution and abundance of chlamydiae

We queried all near full-length 16S rRNA gene sequences (≥1300 nt) present in MAGs from the GEM catalog (*n* = 46) against the IMNGS database^81^ (accessed March 5th, 2018), which systematically collects and preclusters amplicon studies deposited in the short read archive (SRA)^81^. We used a 99% identity cutoff to approximate retrieval of 16S rRNA gene amplicons at the species level to estimate the environmental distribution of chlamydial species with genome representatives. We accepted an SRA sample if at least three reads mapped to a chlamydial 16S rRNA gene query sequence. We classified SRA samples mirroring the IMG/M environmental nomenclature using SRA metadata (https://www.ncbi.nlm.nih.gov/sra)^28^. We tested for overrepresentation of chlamydial families in environments using Fisher’s exact test with the “fisher.test” (“stats“ package) with “alternative = greater” in R version 3.5.1^123^ and corrected *p* values with “BH”^124^ using the R base package function “p.adjust”. We considered *p* values ≤ 0.05 as significant and transformed them into *z*-scores using the “qnorm” function in the stats package.

### Statistical analysis

All statistical tests and data analysis were performed in R version 3.5.1^123^.

### Reporting summary

Further information on research design is available in the Nature Research Reporting Summary linked to this article.

## Supplementary information

Supplementary information

Descriptions of Additional Supplementary Files

Supplementary Data 1

Supplementary Data 2

Supplementary Data 3

Supplementary Data 4

Supplementary Data 5

Supplementary Data 6

Supplementary Data 7

Supplementary Data 8

Supplementary Data 9

Supplementary Data 10

Supplementary Data 11

Supplementary Data 12

Supplementary Data 13

Reporting Summary

## Data Availability

All metagenomic data, bins and annotations are available through the IMG/M portal (https://img.jgi.doe.gov/). Metagenome-assembled genome sequences from the Genomes from Earth’s Microbiomes initiative^25^ are available at https://genome.jgi.doe.gov/GEMs and https://portal.nersc.gov/GEM. Small subunit rRNA gene data used in this study are available via the SILVA database (https://www.arb-silva.de/)^136^ and IMNGS database (https://www.imngs.org/)^81^. Metadata for data used from the IMNGS database can be accessed via the Sequence Read Archive (SRA, https://www.ncbi.nlm.nih.gov/sra)^28^. The collection of MAGs and proteomes used in this study, mapping files (pangenome NOGs, KEGG and Interpro), trimmed alignment files, and tree files are available at zenodo (10.5281/zenodo.4318714). Accession numbers for reference genomes are available in Supplementary Table 2. Additional genome sequences generated in this study have been deposited in GenBank under the accession numbers JAEMUB000000000, JAEMUC000000000, JAEMUD000000000, and JAEMUE000000000.
