# Supplementary material for: Pangenomics reveals alternative environmental lifestyles among chlamydiae
Source: Nat Commun. 2021 Jun 29;12:4021. doi: 10.1038/s41467-021-24294-3 (PMC8242063; doi:10.1038/s41467-021-24294-3)
Supplement: Supplementary file 1 — Supplementary information [file 41467_2021_24294_MOESM1_ESM.pdf]

SUPPLEMENTARY INFORMATION

Pangenomics reveals alternative environmental lifestyles among chlamydiae

Stephan Köstlbacher[1], Astrid Collingro[1], Tamara Halter[1], Frederik Schulz[2], Sean P. Jungbluth[2], and Matthias Horn[1]*

[1] Centre for Microbiology and Environmental Systems Science, University of Vienna, Vienna, Austria

[2] DOE Joint Genome Institute, Berkeley, CA, USA

[3] Current address: Laboratory of Microbiology, Wageningen University and Research, Wageningen, The Netherlands

* Corresponding author: Matthias Horn, matthias.horn@univie.ac.at

Supplementary discussion 1: Patchy nucleotide and TCA metabolism as hallmarks of the accessory genome

Despite a general dependency of all chlamydiae on host resources, previous analysis suggested that environmental representatives retained more complete sets of central metabolic pathways than the pathogenic Chlamydiaceae[1–4]. This trend prevails in our extended dataset comprising a large number of chlamydiae from diverse environments. Consistent with the presence of nucleotide transport proteins for the uptake of ribonucleotides in all chlamydiae, only few genes involved in nucleotide synthesis are part of the phylum core genome. This includes for example the ribonucleotide reductase that catalyzes the reduction of ribonucleotide to deoxyribonucleotides (Figure 3, Supplementary Data 9). Yet, several chlamydiae including MAGs of aquatic origin encode complete *de novo* synthesis pathways for purines or pyrimidines[5–8] (Figure 3, Supplementary Data 9). We observed a similarly patchy distribution for genes functioning in amino acid synthesis, exemplified by the tryptophan synthesis pathway (Figure 3, Supplementary Data 9).

A reduced tricarboxylic acid (TCA) cycle is a hallmark of the Chlamydiaceae[1], which in these pathogens is supplemented by host derived intermediates during intracellular growth to produce biomass[9]. Many environmental chlamydiae encode a complete TCA cycle, but the pathway is truncated or nearly absent in the amoeba-associated *Neochlamydia* species, in members of the Anoxychlamydiales (a clade in the family SM23-39)[5,10], and completely absent in the fish pathogens *Clavichlamydia salmonicola* (Collingro et al, in preparation) and the Parilichlamydiaceae[11] (Figure 3). We found the entire TCA gene set in several environmental MAGs but observed a lack of the majority of the TCA cycle genes in the family MCF-C, and in novel MAGs of the Parachlamydiaceae and Simkaniaceae, several of which were retrieved from marine gutless worms or amoeba isolated from fish gills (Figure 3, Supplementary Data 9). The apparent lack of a TCA cycle in clades of chlamydiae whose members otherwise encode a (nearly) complete pathway points at lineage specific gene loss, potentially associated with major transition events such as the specialization to a new host. In fact, gene loss was proposed as a major driver of genotypic variation, shaping the accessory genome in pathogenic bacteria[12].

Supplementary discussion 2: Motility, mobile genetic elements, and antiviral defense

In addition to a greater metabolic versatility, environmental chlamydiae may encode a number of features unexpected and unusual in the context of the well-conserved chlamydial lifestyle[13]. Some amoeba-associated chlamydiae encode chemosensory systems proposed to regulate unknown cellular functions[2,7,14]. We find evidence for these systems in additional environmental MAGs (Supplementary Data 10). Further, several marine chlamydial SAGs and MAGs encode a semicomplete to complete gene set for a flagellar apparatus, which is generally regulated by chemosensory systems[5,8,15] (Figure 3, Supplementary Data 10). While motility likely represents an ancestral feature of chlamydiae that has been lost in many lineages[5,8], unusual features can also originate from gene gains, which have been shown to play an important role in chlamydial evolution[16–19].

A driver of gene gain by HGT in chlamydiae are plasmids and the conjugative type IV secretion system (T4SS)[2,19,20]. We investigated the presence of the major ATPase VirB4, which is almost ubiquitously found in T4SS[21], and detected homologs in six chlamydial families (Figure 3). Intriguingly, all except one VirB4 homologs (n = 21) are monophyletic and branch as a sister clade to rickettsiae, well-known intracellular alphaproteobacteria including human pathogens and amoeba-associated symbionts[2,19] (Supplementary Fig. 7). The role of the T4SS in the biology of extant chlamydiae is still unclear, but our findings provide further evidence for conjugative T4SS as a mechanism of inter-species HGT among chlamydiae[19].

Chlamydiae were considered to lack the phage defense CRISPR-Cas systems until recently[22]. Yet, a horizontally acquired CRISPR locus was discovered on plasmids of *Protochlamydia naegleriophila* KNic and *Protochlamydia massiliensis* (family Parachlamydiaceae)[23,24]. We screened the MAGs from the GEM catalogue using CRISPRCasFinder[25] and found DNA-targeting type I-C and I-E systems including six to 50 spacers in three genomes (Figure 3, Supplementary Data 11). Nucleotide blast against the NCBI viral refseq did not yield any significant hits (E-value < 0.001) for the spacers to known viral sequences. This illustrates our very limited knowledge about phages targeting chlamydiae, which have so far only been described for chlamydial pathogens in the Chlamydiaceae and sporadically for amoeba symbionts in the Parachlamydiaceae[26,27]. Of note, all chlamydiae with CRISPR-Cas systems stem from freshwater sources (Figure 1), potentially hinting at a higher relevance of phage predation in these environments.

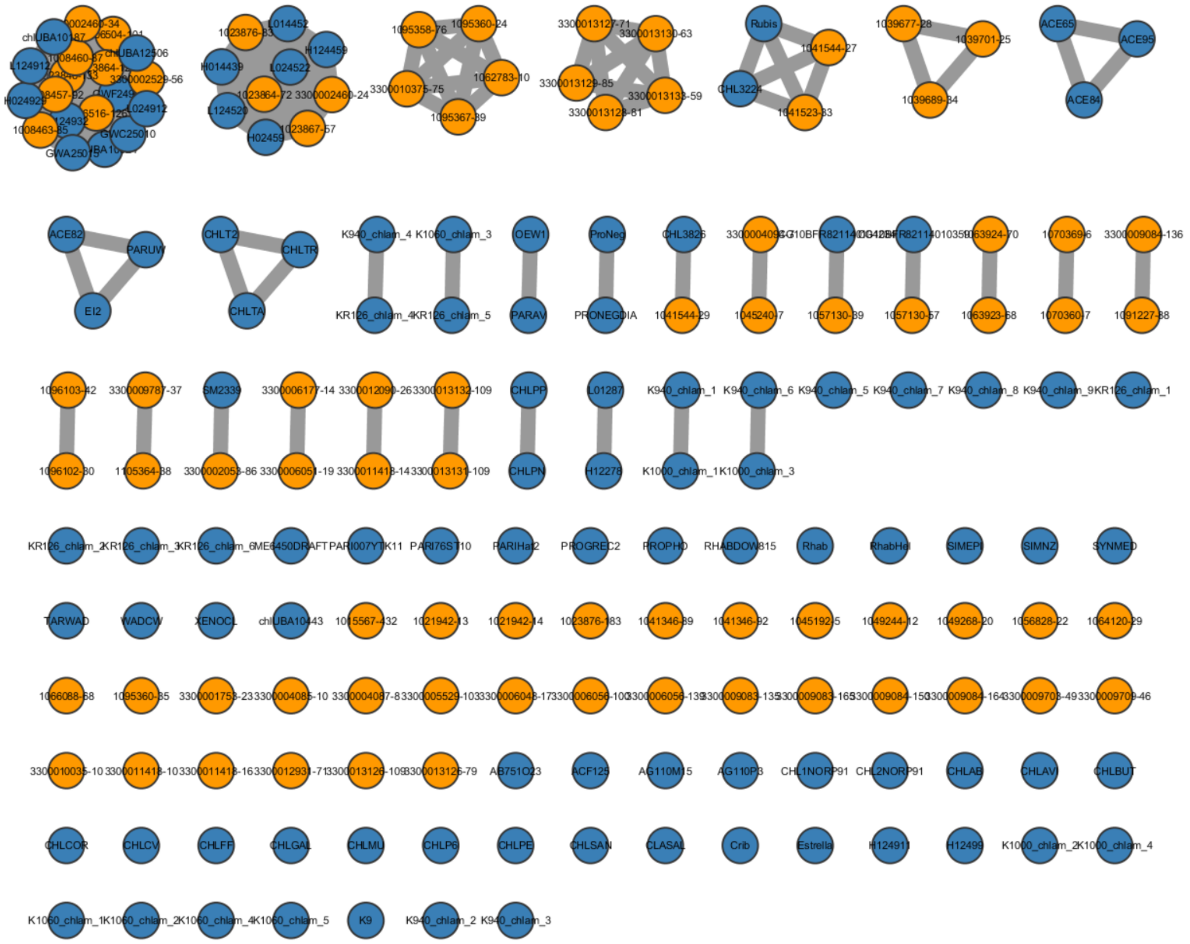

Supplementary Fig. 1: 117 species level clusters formed based on a 95% average nucleotide identity (ANI) cutoff. GEM MAGs are colored in orange and reference genomes in dark blue.

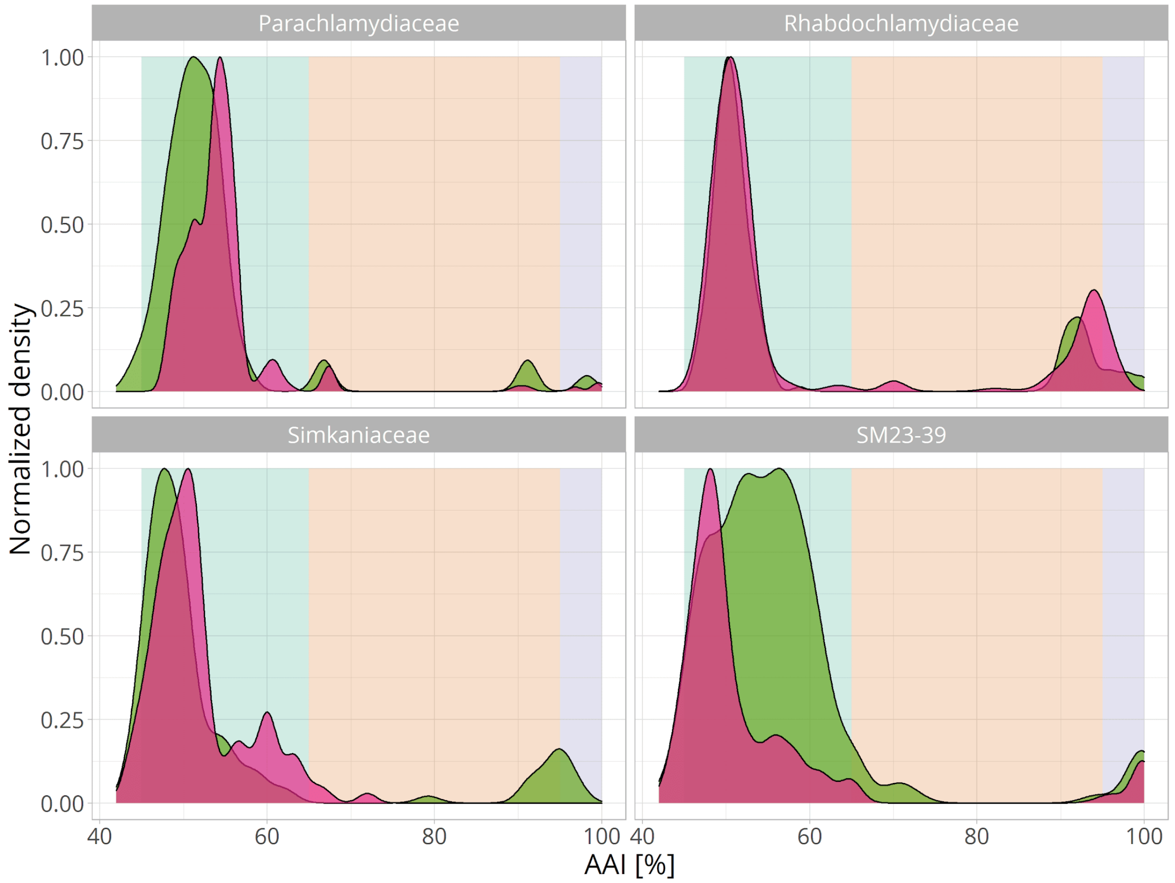

Supplementary Fig. 2: Density distribution of average amino acid identity (AAI) values of GEM MAGs. X-axis indicates AAI [%] and y-axis represents normalized density of AAI values within published chlamydial families. Curve area is colored by GEM AAI to reference (red) or other GEM MAGs (green) in the same described family. Vertical background highlights indicate different genus (45–65% AAI, light green), same genus (65-95% AAI light orange), or same species (> 95% AAI light purple) as defined by[28].

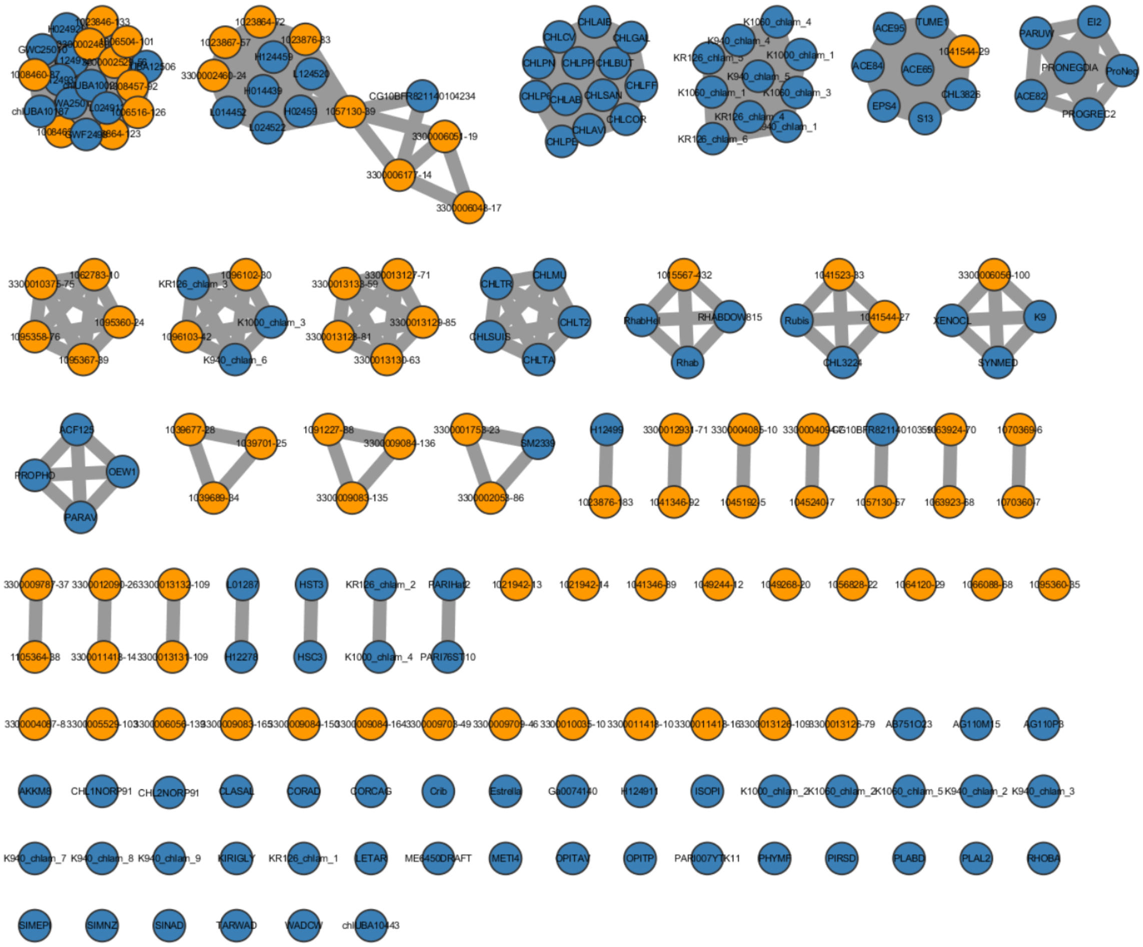

Supplementary Fig. 3: 94 genus level clusters formed based on a 65% average amino acid identity (AAI) cutoff. GEM MAGs are colored in orange and reference genomes in dark blue.

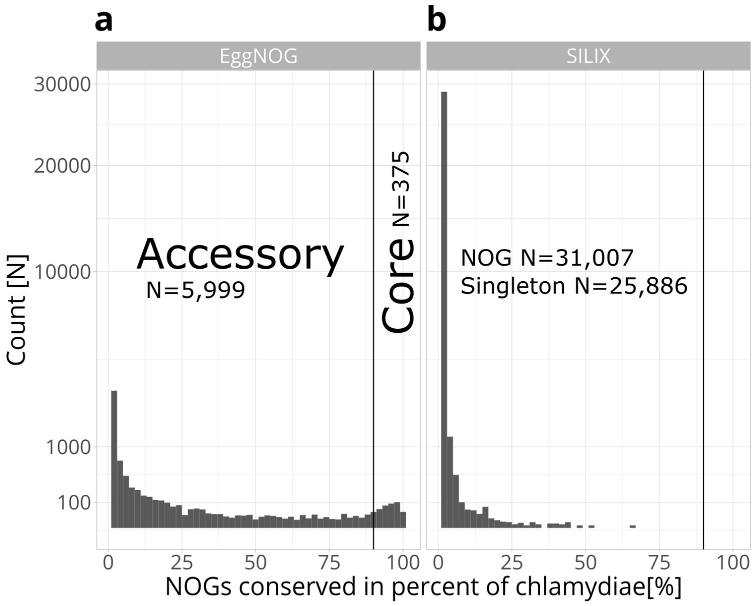

Supplementary Fig. 4: Chlamydial pangenome conservation of non-supervised orthologous groups (NOGs). NOGs were classified as core (conserved in > 90% of genomes), accessory genome (conserved in ≤90%). (**a**) NOGs derived from mapping to EggNOG 4.5 and (**b**) *de novo* clustering with SiLIX of unmapped genes.

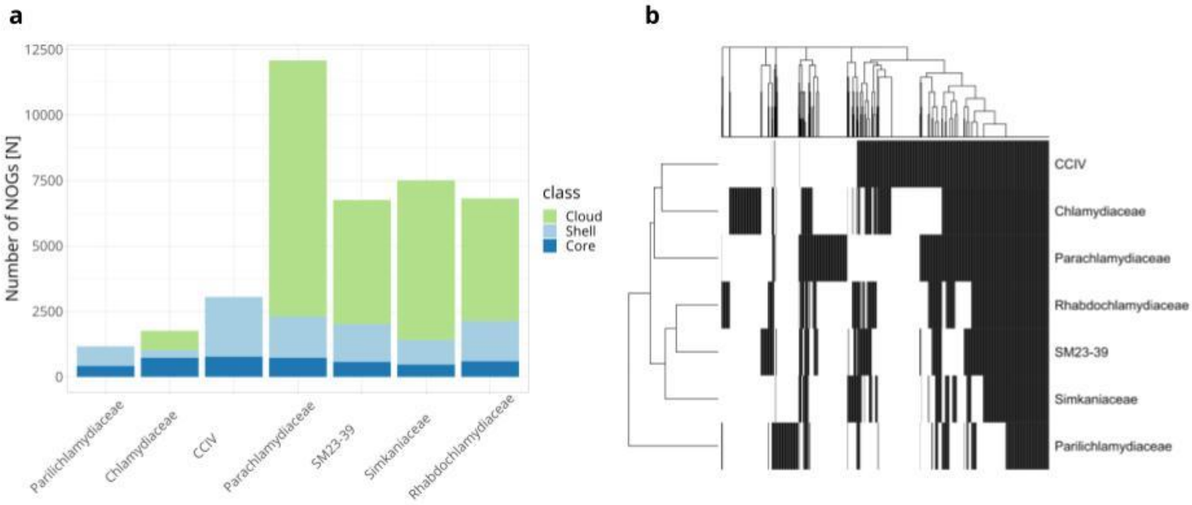

Supplementary Fig. 5: Stable core genome and variable accessory genome sizes in chlamydial families with more than three members. (**a**) Core and accessory genome sizes of chlamydial families represented by at least three high quality genomes. (**b**) Clustering of the pangenome of chlamydial families based on presence/absence of genes.

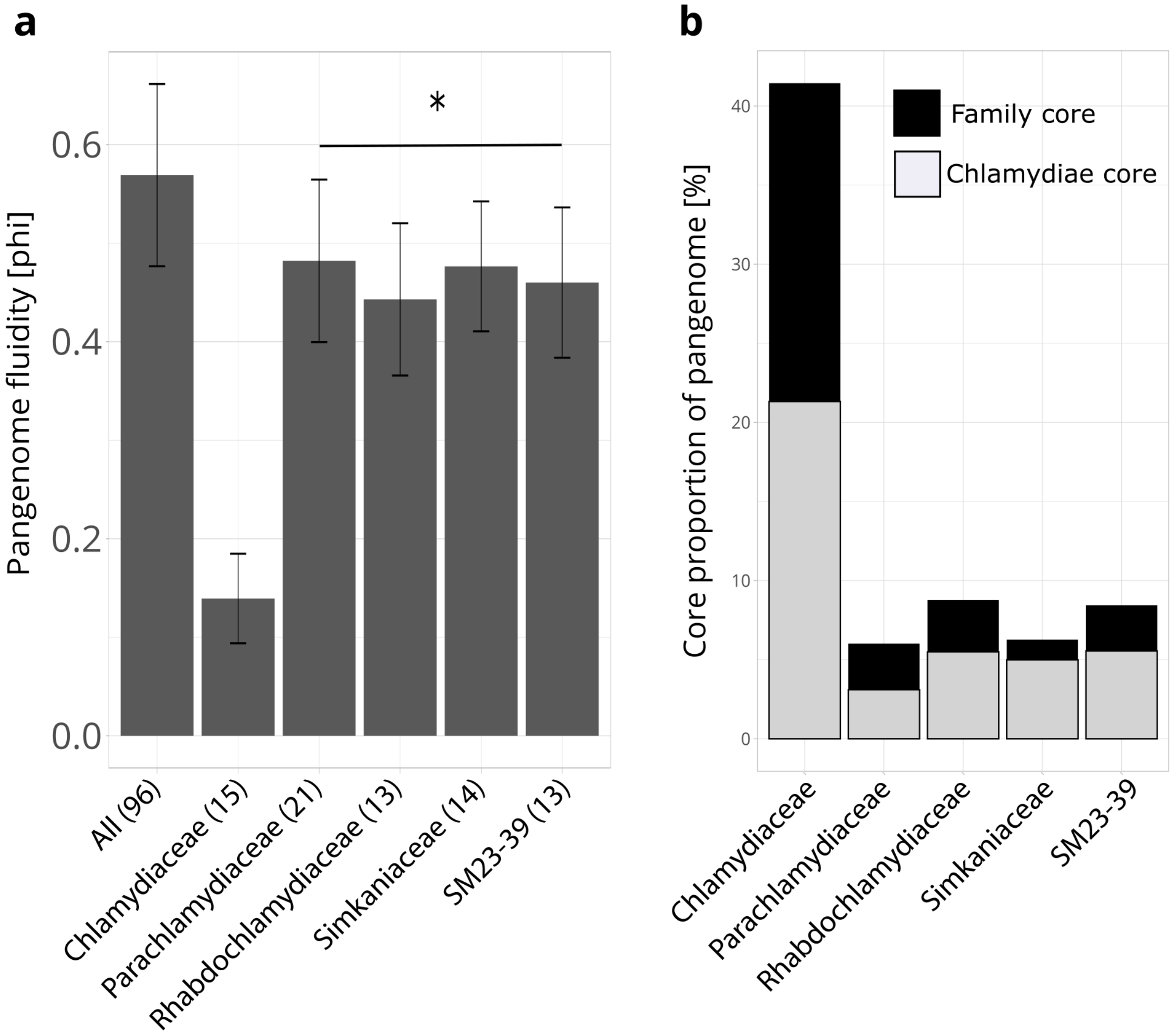

Supplementary Fig. 6: (a) Genomic fluidity of chlamydial families with at least 10 members. Bars represent mean genome fluidity between family representative genomes +/- standard deviation as error bars. Number of representative genomes used for comparison indicated in brackets next to the family label. 100 random genome pairs were compared to calculate the mean fluidity. The asterisk denotes families with a significantly different fluidity from Chlamydiaceae (p-values ≤ 0.05) based on two-sample t-tests (FDR adjusted p-values from left to right starting with Parachlamydiaceae: 3.8^{-13}, 3.9^{-10}, 8.0^{-11}, 5.4^{-10}). **(b)** Proportion of the core genome (chlamydiae- and family-specific, respectively) of the pangenome**.**

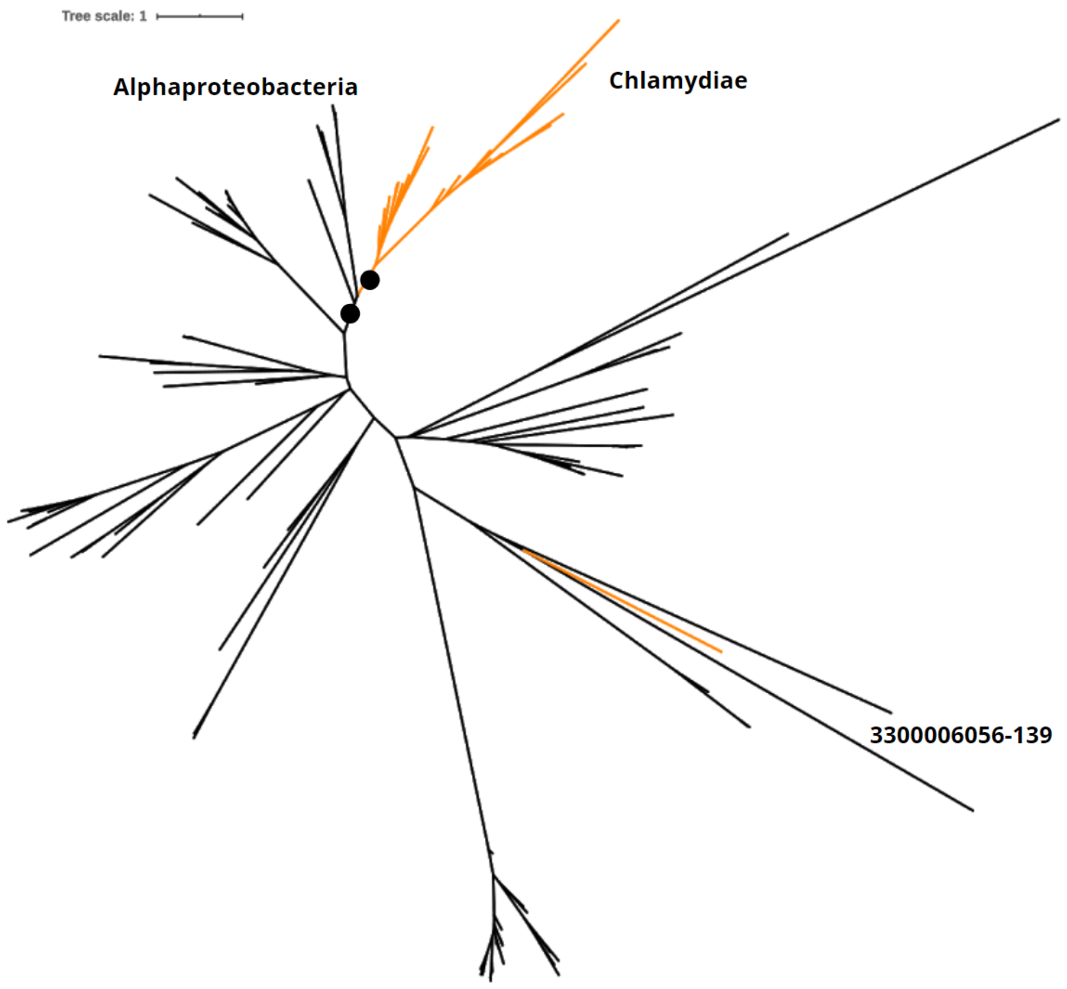

Supplementary Fig. 7: Maximum likelihood phylogenetic tree of ENOG4107S2X (VirB4) with chlamydial sequences. A large chlamydial VirB4 homologous clade (improved ultrafast bootstrap 98.2%, SH like test 97%) is sister to an alphaproteobacterial clade (improved ultrafast bootstrap 95.4%, SH like test 97%). Maximum likelihood tree was inferred under LG+C50+G+F model with 1,000 improved ultrafast bootstraps and 1,000 replicates of the SH-like approximate likelihood ratio test.

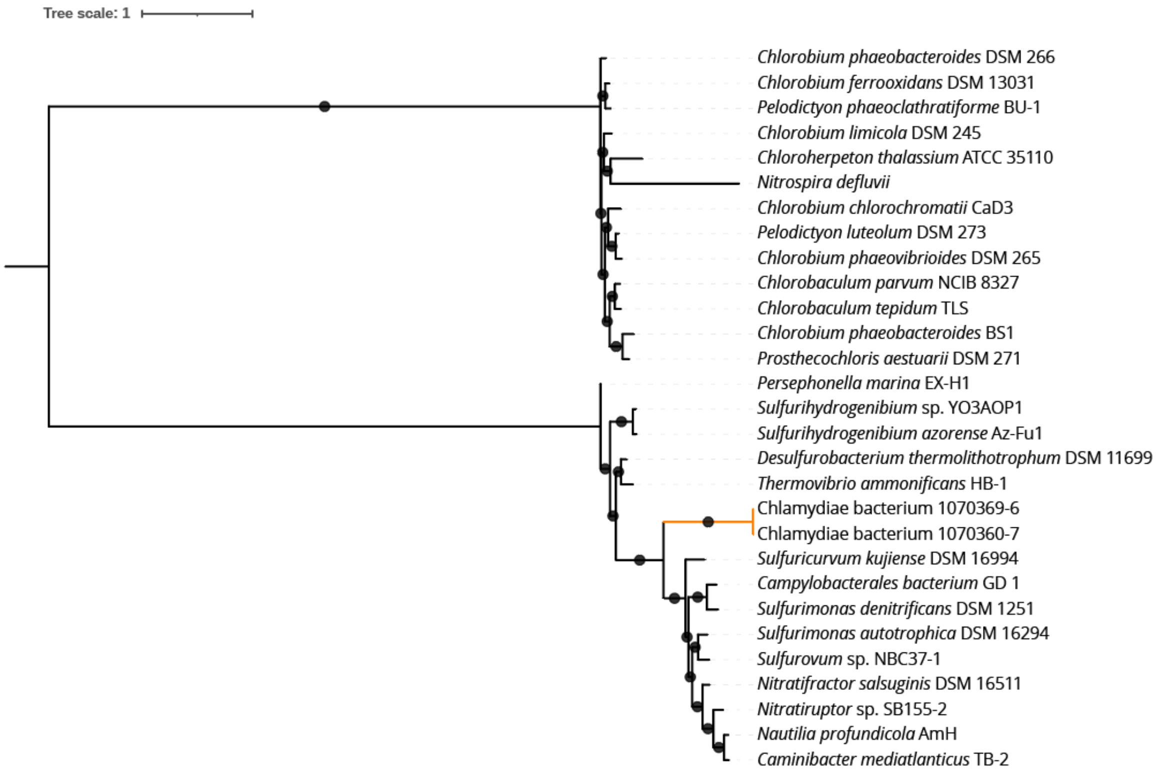

Supplementary Fig. 8: Maximum likelihood phylogenetic tree of ENOG4105C63 (AclA) with chlamydial sequences. Chlamydiae are monophyletic with a clade of campylobacterotal AclA (improved ultrafast bootstrap 100%, SH like test 100%). Maximum likelihood tree was inferred under LG+C20+G+F model with 1,000 improved ultrafast bootstraps and 1,000 replicates of the SH-like approximate likelihood ratio test. Filled circles at nodes indicate a bootstrap support > 95%.

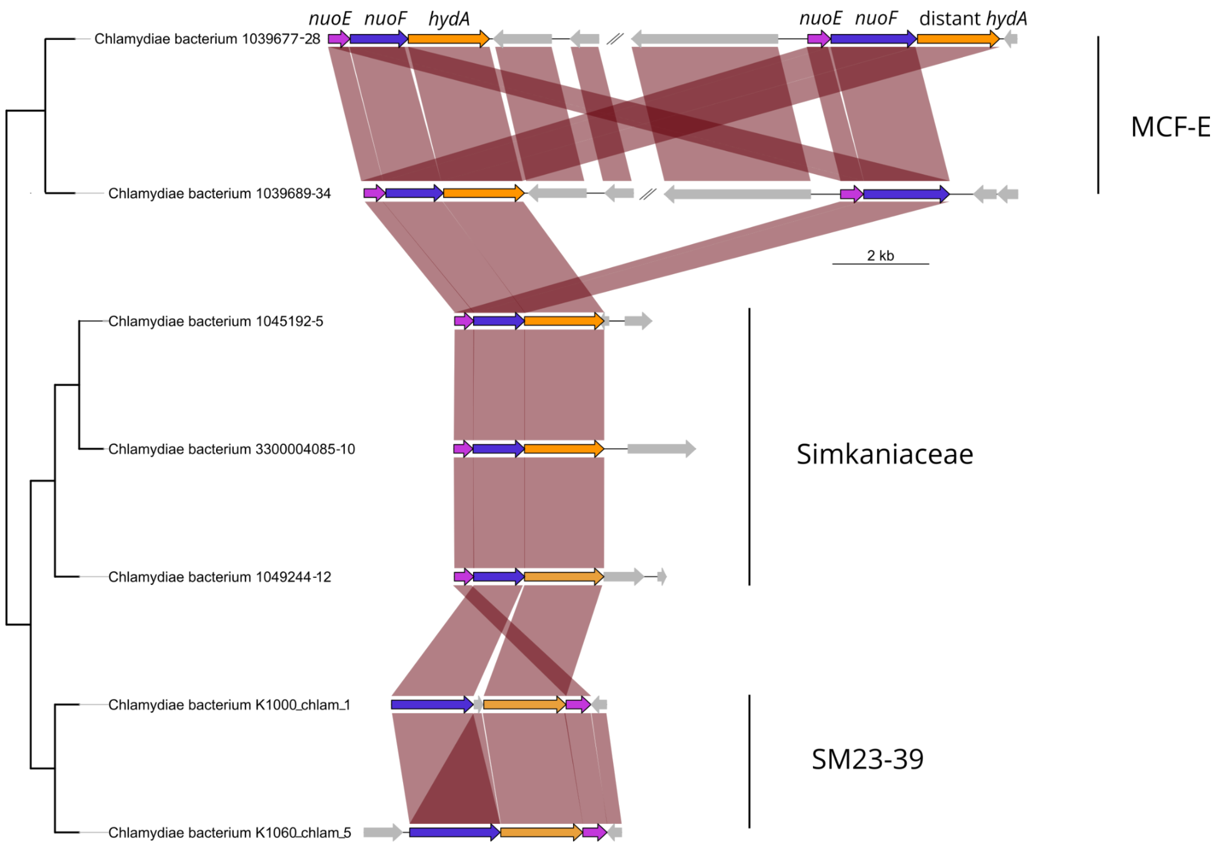

Supplementary Fig. 9: Gene cluster structure of representatives of group A [FeFe]-hydrogenases encoded in members of the families SM23-39, Simkaniaceae, and MCF-E. The second gene cluster in MCF-E contains the distant copy of *hydA*. Comparisons are ordered according to phylogenomic species tree. Red bands indicate genes belonging to the respective NOG.

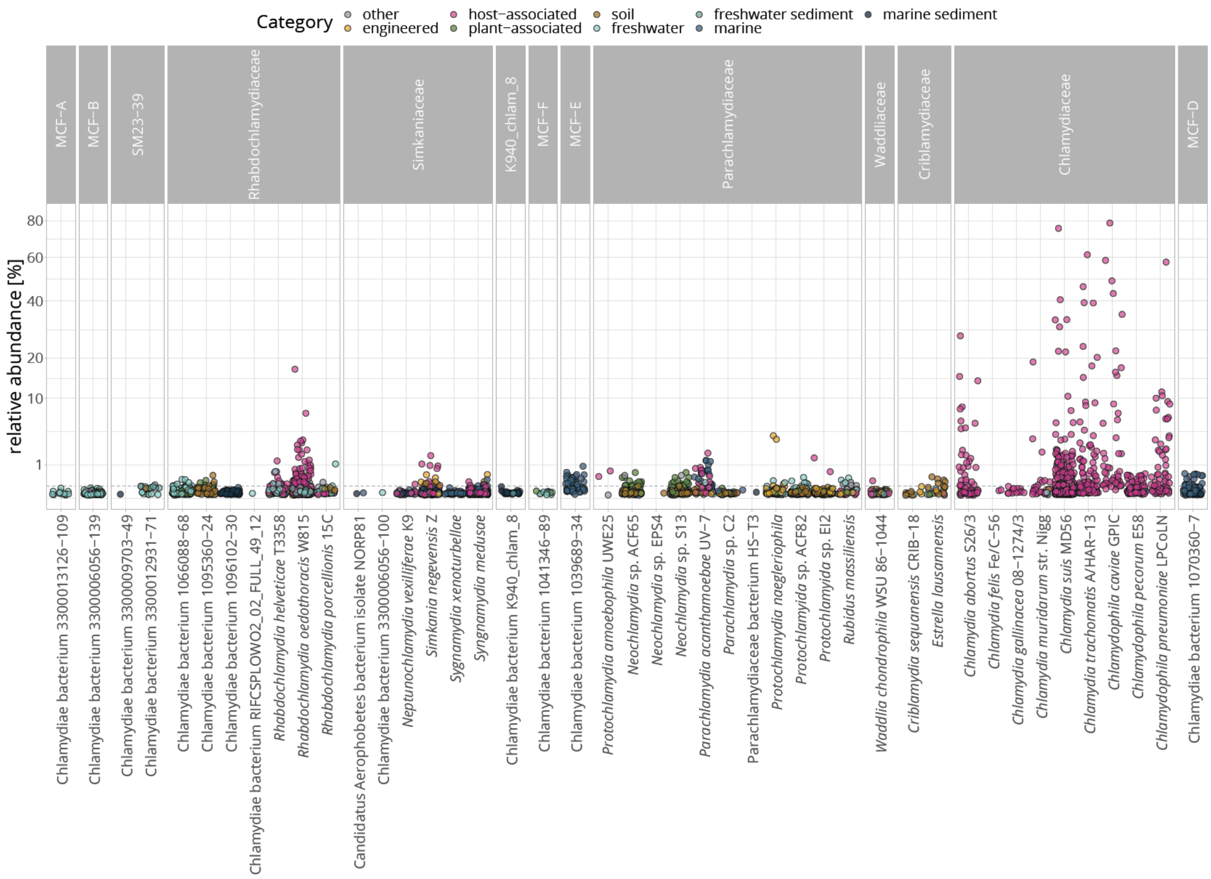

Supplementary Fig. 10: Relative abundance of chlamydial 16S rRNA gene amplicons in SRA samples per species representative with a sequenced genome.

Supplementary references

1. Omsland, A., Sixt, B. S., Horn, M. & Hackstadt, T. Chlamydial metabolism revisited: interspecies metabolic variability and developmental stage-specific physiologic activities. *FEMS Microbiol. Rev.* **38**, 779–801 (2014).
2. Collingro, A. *et al.* Unity in Variety--The Pan-Genome of the Chlamydiae. *Molecular Biology and Evolution* **28**, 3253–3270 (2011).
3. Bertelli, C. *et al.* The Waddlia Genome: A Window into Chlamydial Biology. *PLoS ONE* **5**, e10890 (2010).
4. Bertelli, C. *et al.* Sequencing and characterizing the genome of Estrella lausannensis as an undergraduate project: training students and biological insights. *Front. Microbiol.* **6**,

101 (2015).

5. Dharamshi, J. E. *et al.* Marine Sediments Illuminate Chlamydiae Diversity and Evolution. *Curr. Biol.* **30**, 1032–1048.e7 (2020).
6. Bertelli, C. *et al.* The Waddlia Genome: A Window into Chlamydial Biology. *PLoS ONE* **5**, e10890 (2010).
7. Bertelli, C. *et al.* Sequencing and characterizing the genome of Estrella lausannensis as an undergraduate project: training students and biological insights. *Front. Microbiol.* **6**, 101 (2015).
8. Collingro, A. *et al.* Unexpected genomic features in widespread intracellular bacteria: evidence for motility of marine chlamydiae. *ISME J.* **11**, 2334–2344 (2017).
9. Mehlitz, A. *et al.* Metabolic adaptation of Chlamydia trachomatis to mammalian host cells. *Mol. Microbiol.* **103**, 1004–1019 (2017).
10. Ishida, K. *et al.* Amoebal endosymbiont Neochlamydia genome sequence illuminates the bacterial role in the defense of the host amoebae against Legionella pneumophila. *PLoS One* **9**, e95166 (2014).
11. Taylor-Brown, A. *et al.* Metagenomic Analysis of Fish-Associated Ca. Parilichlamydiaceae Reveals Striking Metabolic Similarities to the Terrestrial Chlamydiaceae. *Genome Biol. Evol.* **10**, 2587–2595 (2018).
12. Bolotin, E. & Hershberg, R. Gene Loss Dominates As a Source of Genetic Variation within Clonal Pathogenic Bacterial Species. *Genome Biol. Evol.* **7**, 2173–2187 (2015).
13. Collingro, A., Köstlbacher, S. & Horn, M. Chlamydiae in the Environment. *Trends Microbiol.* (2020) doi:10.1016/j.tim.2020.05.020.
14. Bertelli, C., Goesmann, A. & Greub, G. Criblamydia sequanensis Harbors a Megaplasmid Encoding Arsenite Resistance. *Genome Announc.* **2**, (2014).
15. Tully, B. J., Graham, E. D. & Heidelberg, J. F. The reconstruction of 2,631 draft metagenome-assembled genomes from the global oceans. *Sci Data* **5**, 170203 (2018).

16. Kim, H., Kwak, W., Yoon, S. H., Kang, D.-K. & Kim, H. Horizontal gene transfer of Chlamydia: Novel insights from tree reconciliation. *PLoS One* **13**, e0195139 (2018).
17. Domman, D. *et al.* Massive expansion of Ubiquitination-related gene families within the Chlamydiae. *Mol. Biol. Evol.* **31**, 2890–2904 (2014).
18. Kamneva, O. K., Knight, S. J., Liberles, D. A. & Ward, N. L. Analysis of genome content evolution in pvc bacterial super-phylum: assessment of candidate genes associated with cellular organization and lifestyle. *Genome Biol. Evol.* **4**, 1375–1390 (2012).
19. Köstlbacher, S., Collingro, A., Halter, T., Domman, D. & Horn, M. Coevolving Plasmids Drive Gene Flow and Genome Plasticity in Host-Associated Intracellular Bacteria. *Current Biology* **31**, 346–357.e3 (2021).
20. Greub, G., Collyn, F., Guy, L. & Roten, C.-A. A genomic island present along the bacterial chromosome of the Parachlamydiaceae UWE25, an obligate amoebal endosymbiont, encodes a potentially functional F-like conjugative DNA transfer system. *BMC Microbiol.* **4**, 48 (2004).
21. Guglielmini, J. *et al.* Key components of the eight classes of type IV secretion systems involved in bacterial conjugation or protein secretion. *Nucleic Acids Research* **42**, 5715–5727 (2014).
22. Burstein, D. *et al.* Major bacterial lineages are essentially devoid of CRISPR-Cas viral defence systems. *Nat. Commun.* **7**, 10613 (2016).
23. Bertelli, C. *et al.* CRISPR System Acquisition and Evolution of an Obligate IntracellularChlamydia-Related Bacterium. *Genome Biology and Evolution* **8**, 2376–2386 (2016).
24. Benamar, S. *et al.* Developmental Cycle and Genome Analysis of Protochlamydia massiliensis sp. nov. a New Species in the Parachlamydiacae Family. *Front. Cell. Infect. Microbiol.* **7**, (2017).
25. Couvin, D. *et al.* CRISPRCasFinder, an update of CRISRFinder, includes a portable

version, enhanced performance and integrates search for Cas proteins. *Nucleic Acids Res.* **46**, W246–W251 (2018).

26. Śliwa-Dominiak, J., Suszyńska, E., Pawlikowska, M. & Deptuła, W. Chlamydia bacteriophages. *Arch. Microbiol.* **195**, 765–771 (2013).

27. Corsaro, D., Müller, K.-D., Wingender, J. & Michel, R. 'Candidatus Mesochlamydia elodeae' (Chlamydiae: Parachlamydiaceae), a novel chlamydia parasite of free-living amoebae. *Parasitology Research* **112**, 829–838 (2013).

28. Konstantinidis, K. T., Rosselló-Móra, R. & Amann, R. Uncultivated microbes in need of their own taxonomy. *ISME J.* **11**, 2399–2406 (2017).
