# Supplementary material for: Pangenomics reveals alternative environmental lifestyles among chlamydiae
Source: Nat Commun. 2021 Jun 29;12:4021. doi: 10.1038/s41467-021-24294-3 (PMC8242063; doi:10.1038/s41467-021-24294-3)
Supplement: Supplementary file 2 — Descriptions of Additional Supplementary Files [file 41467_2021_24294_MOESM2_ESM.pdf]

Descriptions of Additional Supplementary Files

Supplementary Data 1
Description: Chlamydial MAGs and genome information.

Supplementary Data 2
Description: PVC reference dataset.

Supplementary Data 3
Description: Phylogenetic distance (PD) of known chlamydiae versus chlamydial MAGs in species tree, and phylogenetic gain (PG) by MAGs.

Supplementary Data 4
Description: Environmental origin of chlamydial genomes. Supplementary Data 5. Phylogenetic gain by GEM MAGs per environment.

Supplementary Data 6
Description: GTDB-Tk output for chlamydial genomes.

Supplementary Data 7
Description: Adjusted taxonomic affiliation of chlamydial genomes.

Supplementary Data 8
Desfription: EggNOG NOGs per genome of the dereplicated dataset.

Supplementary Data 9
Description: Presence of selected metabolic pathway in dereplicated chlamydial MAGs.

Supplementary Data 10
Description: Chemotaxis in chlamydiae.

Supplementary Data 11
Description: CRISPR-Cas systems in chlamydiae.

Supplementary Data 12
Description: Chlamydial hydrogenases. Supplementary Data 13. Relative abundance of chlamydiae in IMNGS samples.
