# Supplementary material for: Pangenomics reveals alternative environmental lifestyles among chlamydiae
Source: Nat Commun. 2021 Jun 29;12:4021. doi: 10.1038/s41467-021-24294-3 (PMC8242063; doi:10.1038/s41467-021-24294-3)
Supplement: Supplementary file 16 — Reporting Summary [file 41467_2021_24294_MOESM16_ESM.pdf]

nature research

Corresponding author(s): Matthias Horn

Last updated by author(s): May 4, 2021

Reporting Summary

Nature Research wishes to improve the reproducibility of the work that we publish. This form provides structure for consistency and transparency in reporting. For further information on Nature Research policies, see our Editorial Policies and the Editorial Policy Checklist.

Statistics

For all statistical analyses, confirm that the following items are present in the figure legend, table legend, main text, or Methods section.

n/a	Confirmed	
☒	☐	The exact sample size (*n*) for each experimental group/condition, given as a discrete number and unit of measurement
☒	☐	A statement on whether measurements were taken from distinct samples or whether the same sample was measured repeatedly
☐	☒	The statistical test(s) used AND whether they are one- or two-sided<br>*Only common tests should be described solely by name; describe more complex techniques in the Methods section.*
☒	☐	A description of all covariates tested
☐	☒	A description of any assumptions or corrections, such as tests of normality and adjustment for multiple comparisons
☐	☒	A full description of the statistical parameters including central tendency (e.g. means) or other basic estimates (e.g. regression coefficient) AND variation (e.g. standard deviation) or associated estimates of uncertainty (e.g. confidence intervals)
☐	☒	For null hypothesis testing, the test statistic (e.g. *F*, *t*, *r*) with confidence intervals, effect sizes, degrees of freedom and *P* value noted<br>*Give P values as exact values whenever suitable.*
☒	☐	For Bayesian analysis, information on the choice of priors and Markov chain Monte Carlo settings
☒	☐	For hierarchical and complex designs, identification of the appropriate level for tests and full reporting of outcomes
☒	☐	Estimates of effect sizes (e.g. Cohen's *d*, Pearson's *r*), indicating how they were calculated

Our web collection on statistics for biologists contains articles on many of the points above.

Software and code

Policy information about availability of computer code

Data collection	BBMap v35.43, FastQC v0.11.4, SPAdes v3.5.0, prokka v1.14, CheckM v1.1.2
Data analysis	QUAST v5.0.2; prokka v1.14; CheckM v1.1.2; IQ-TREE 1.6.2; Interactive Tree Of Life v4; GenomeTreeTk v0.1.6; dRep v1.4.3; GTDB-Tk v0.3.3; emapper v1.0.1; SiLiX v1.2.11; R v3.5.1; micropan v2.1; GhostKOALA v2.2; InterProScan v5.35-74.0; MacSyFinder v1.0.5; CRISPRCasFinder v2.0.2; USEARCH v11.0.667; FastANI v1.3; BMGE v1.12; trimAl; booster; Cytoscape v3.7.0; ggplot2; ggraph; genoplotR v0.8.9

For manuscripts utilizing custom algorithms or software that are central to the research but not yet described in published literature, software must be made available to editors and reviewers. We strongly encourage code deposition in a community repository (e.g. GitHub). See the Nature Research guidelines for submitting code & software for further information.

Data

Policy information about availability of data

All manuscripts must include a data availability statement. This statement should provide the following information, where applicable:

- Accession codes, unique identifiers, or web links for publicly available datasets
- A list of figures that have associated raw data
- A description of any restrictions on data availability

All metagenomic data, bins and annotations are available through the IMG/M portal (https://img.jgi.doe.gov/). Metagenome-assembled genome sequences from the Genomes from Earth's Microbiomes initiative are available at https://genome.jgi.doe.gov/GEMs and https://portal.nersc.gov/GEM. Small subunit rRNA gene data used in this study are available via the SILVA database (https://www.arb-silva.de/) and IMNGS database (https://www.imngs.org/). Metadata for data used from the IMNGS database can be accessed via the Sequence Read Archive (SRA, https://www.ncbi.nlm.nih.gov/sra). The collection of MAGs and proteomes used in this study, mapping files (pangenome NOGs, KEGG and Interpro), alignment files, and tree files are available at zenodo (https://doi.org/10.5281/zenodo.4318714). Accession numbers for reference genomes are available in Supplementary Table 2. Additional genome sequences generated in this study have been deposited in

GenBank under the accession numbers JAEMUB000000000, JAEMUC000000000, JAEMUD000000000, and JAEMUE000000000.

Field-specific reporting

Please select the one below that is the best fit for your research. If you are not sure, read the appropriate sections before making your selection.

- [x] Life sciences
- [ ] Behavioural & social sciences
- [ ] Ecological, evolutionary & environmental sciences

For a reference copy of the document with all sections, see nature.com/documents/nr-reporting-summary-flat.pdf

Life sciences study design

All studies must disclose on these points even when the disclosure is negative.

Sample size	Our initial genome sequence dataset comprised 82 metagenome-assembled genomes (MAGs) of the GEM catalogue. We added four additional draft genome sequences, 106 publicly available reference genomes, and 15 genome sequences as outgroup. This dataset represents the current genome diversity of members of the Phylum Chlamydiae, enabling detailed comparative genomic and phylogenetic analyses.
Data exclusions	After initial phylogenetic and phylogenomic analysis, we excluded 79 genome sequences from a more detailed downstream pangenome analysis based on quality criteria (completeness < 85%, contamination >= 5%), and to reduce redundancy. Functional gene phylogenetic analysis always included all genomes.
Replication	All analyses were performed once, but can be reproduced with the parameters and program versions provided in the methods section.
Randomization	We used 100 random samples for the computation of genome fluidity. Otherwise all samples were analyzed, therefore randomization was not necessary.
Blinding	Blinding was not relevant here, as no direct comparisons or conclusions were drawn from how samples were treated.

Reporting for specific materials, systems and methods

We require information from authors about some types of materials, experimental systems and methods used in many studies. Here, indicate whether each material, system or method listed is relevant to your study. If you are not sure if a list item applies to your research, read the appropriate section before selecting a response.

Materials & experimental systems

n/a	Involved in the study	
x		Antibodies
x		Eukaryotic cell lines
x		Palaeontology and archaeology
x		Animals and other organisms
x		Human research participants
x		Clinical data
x		Dual use research of concern

Methods

n/a	Involved in the study	
x		ChIP-seq
x		Flow cytometry
x		MRI-based neuroimaging
